# The Impact of Meteorological Conditions and Agricultural Waste Burning on PM Levels: A Case Study of Avellino (Southern Italy)

**DOI:** 10.3390/ijerph191912246

**Published:** 2022-09-27

**Authors:** Vincenzo Capozzi, Letizia Raia, Viviana Cretella, Carmela De Vivo, Raffaele Cucciniello

**Affiliations:** 1 Department of Science and Technology, University of Naples “Parthenope”, Centro Direzionale di Napoli—Isola C4, 80143 Naples, Italy; 2 Department of Chemistry and Biology “Adolfo Zambelli”, University of Salerno, Via Giovanni Paolo II, 132, 84084 Fisciano, Italy

**Keywords:** PM, air quality, thermal inversion, Avellino, biomass burning

## Abstract

In this work, the effect of the meteorological conditions and the agricultural waste burning on PM air pollution levels has been investigated in the city of Avellino, located in the Sabato Valley (southern Italy). Avellino has been described among the most polluted towns in Italy in terms of particulate matter (PM) during the last 10 years. The main aim of this study was to analyze the air quality data collected in Avellino and its surroundings during September 2021. In this period, the air quality in the Sabato Valley has been adversely affected by agricultural practices, which represent a significant source of PM. The impact of agricultural waste burning on PM levels in Avellino has been determined through an integrated monitoring network, consisting of two fixed urban reference stations and by several low-cost sensors distributed in the Sabato Valley. In the considered period, the two reference stations recorded several exceedances of the daily average PM_10_ legislative limit value (50 µg m^−3^) in addition to high concentrations of PM_2.5_. Moreover, we provide a detailed description of the event that took place on 25 September 2021, when the combined effect of massive agricultural practices and very stable atmospheric conditions produced a severe pollution episode. Results show PM exceedances in Avellino concurrent with high PM values in the areas bordering the city due to agricultural waste burning and adverse meteorological conditions, which inhibit PM dispersion in the atmosphere.

## 1. Introduction

In order to mitigate the pollution from particulate matter (PM), it is essential to assess the contribution of the sources that modulate the atmospheric aerosol levels. This approach can help in adopting targeted strategies, and many studies rely on this source apportionment to fulfill this objective [1,2,3]. The scientific community, moving from detailed and consistent evaluations, plays a central role addressing policymakers [4]. The banning of biomass burning and vehicular traffic and the interruption of industrial activities in concomitance with severe meteorological conditions that inhibits pollutant dispersion in the atmosphere are some adopted practices following an integrated approach between policy and the scientific community. Several studies focused on the risk to human health associated with particulate pollution in the light of recent demonstrated correlations between PM pollution and COVID-19 diffusion [5]. It is well known that aerosol chemical characteristics depend on sources and weather conditions [6,7,8]. In this context, biomass and agricultural waste burning have been identified as significant sources of PM, especially in rural areas, with negative health effects in respect of cardiovascular and respiratory diseases [9].

It is widely accepted that meteorological conditions, involving both large-scale atmospheric circulation patterns as well as local-scale boundary layer processes, are one of the primary drivers of PM variability [10,11,12]. The incidence of atmospheric parameters on air quality is quite complex and may be summarized as follows. Wind speed and direction exert a major role in controlling the dispersion and the advection of the pollutants. In other words, winds can carry PM away from their original sources, causing a local decrease in surface particle concentration, but at the same time they can disperse them in other locations, causing a worsening in air quality conditions hundreds or thousands of kilometers away from the pollution source. The moisture level in the atmosphere is correlated with processes favorable to secondary particle formation [13], whereas precipitation is known to be a very efficient atmospheric aerosol sink through the well-known process of wet deposition. The daily evolution of such atmospheric variables, modulated by synoptic-scale conditions and by solar radiation, is strictly related to the dynamics of the planetary boundary layer (PBL), which is a shallow layer near the ground that controls the destiny, in terms of mixing, dispersion, transport, transformation, and deposition, of any pollutants emitted into the atmosphere.

In recent years and decades, different research activities have been devoted to the study of the relationship between meteorological conditions and severe pollution events, as testified by many works carried out in China [14,15,16], as well as in Europe and Malaysia [17,18]. In the Italian context, worthy of mention is the analysis of high PM_10_ levels in the metropolitan area of Naples (southern Italy) carried out in [19]. According to the findings of this work, bad air quality in Naples occurs in conjunction with weak wind intensity, thermal inversion and absence of rainfall for at least seven days, mainly during the winter season. More recently, an analysis of the PM_10_ concentration in the urban area of Rome has been performed [20]. From this work, it emerges that in the case of thermal inversion and low wind speed, both horizontal and vertical dispersion of pollutants are very limited. Conversely, the best case for air quality and ventilation occurs when winds blow from the north with a speed exceeding 4 m s^−1^ for at least 24 h, causing the advection of cold and dry air masses. Other interesting evidence about the incidence of synoptic circulation and other PBL features on PM_10_ levels have been provided in [21]. According to this study, based on numerical simulations performed for Changsha city (one of the most polluted areas in China), variation in PM_10_ concentrations is strictly dependent on both wind speed and mixed layer depth.

This work is focused on Avellino, a small city located in the Sabato Valley (Campania Region, southern Italy), for two main reasons. Firstly, Avellino is considered among the worst Italian cities for PM pollution in terms of number of PM_10_ exceedances and for the PM_10_ annual average of 30 μg m^−3^ (twice the guideline value set by the World Health Organization, i.e., 15 μg m^−3^) [22,23,24,25], and only one manuscript has been published on this important issue in this city [26].

According to European and Italian legislation [25], the daily average concentration of PM_10_ threshold (i.e., 50 µg m^−3^) can be exceeded at most 35 days during a year. As a matter of fact, notwithstanding the absence of relevant industrial activities, in Avellino the number of yearly exceedances of PM_10_ has been greater than the legislative threshold for the last nine consecutive years. More specifically, the Agenzia Regionale per la Protezione Ambientale in Campania (hereafter ARPAC), which is the institution that controls the state of the environment, has reported up to 51 exceedances of daily average PM_10_ values in Avellino during 2021 and up to 78 in 2020 [26]. A previous work focused on the area of Avellino has considered the effect of the 2020 lockdown on air quality, showing no relevant influence of COVID-19 countermeasures on PM levels [26]. These results have highlighted the non-predominant role of vehicular traffic compared to other PM sources.

Starting from these results, the scope of the present work was to evaluate the effect of agricultural waste burning and meteorological conditions on air quality observed in Avellino and neighboring areas during September 2021. This aspect was investigated using data obtained by the two monitoring stations located in Avellino, managed by ARPAC, and by an additional network, named AURA—Protegge il nostro respiro (hereafter AURA), which is the fruit of a citizen-science initiative coordinated by local environmental associations. These data, properly integrated with ancillary meteorological measurements, allow evaluation of the PM levels in the areas surrounding Avellino and to analyze their relationship with atmospheric conditions.

## 2. Materials and Methods

### 2.1. Study Area

**Figure 1:**
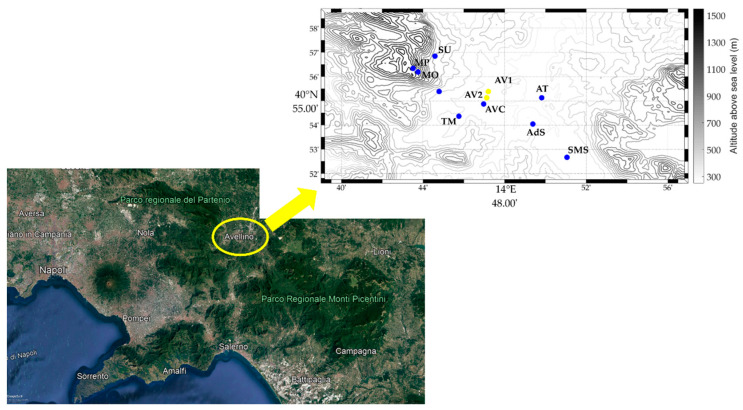
Study area (**lower panel**) and colocation of ARPAC and AURA monitoring stations (**upper panel**). The yellow circles indicate the ARPAC stations, whereas the blue circles refer to the AURA network.

This study was focused on air quality evaluation in Avellino, a small city (40°54′55″ N, 14°47′23″ E, 348 m above sea level covering an area of 30 km^2^) of 53,100 inhabitants (data 2020) located in inland Campania Region (southern Italy) in a valley crossed by the Sabato river, named Sabato Valley. As sketched by Figure 1, the Sabato Valley is closed in the northwestern direction by the Partenio mountains (1573 m above sea level, hereafter asl) and in the eastern and southern directions by the Picentini reliefs (whose maximum height is 1806 m asl). On the southwestern and western sides, it is bounded by the modest reliefs of Vallo di Lauro, whose height ranges between 850 and 1106 m asl. In the northern and northeastern directions, there are no relevant orographic features. In the southern direction, the Sabato Valley is connected to the Gulf of Salerno through the Irno Valley. As reported in previous work, Avellino is a small city without important industrial plants and has mainly light traffic. The use of domestic heating systems is limited from November/December to the end of April [26]. Avellino is in the flat part of a basin surrounded by various mountainous aggregates characterized by important agricultural activities dedicated to the production of hazelnuts, chestnuts, and wine. Therefore, the abovementioned agricultural activities are PM sources to consider due to the final treatment that involves the burning of leaves.

#### Meteorological Characterization

The meteorological regime of this area is very complex, due to its geographical position, barycentric with respect to the Mediterranean Sea and the Balkan region. On average, the Sabato Valley receives a yearly rainfall of about 1350 mm, distributed in 110 days. The precipitation events are mainly triggered by atmospheric transients developing from the polar front, and in the summer season by local convective instability, often enhanced by the orographic features. According to the anemometric data collected in Avellino city in the last 15 years (2007–2021), the average wind speed is 2.2 m s^−1^ on a yearly basis and presents a maximum between late winter and early spring (3.0 m s^−1^ in March) and a minimum in the summer season (1.7 m s^−1^ in August). From October to May, the wind regime is mainly modulated by synoptic-scale circulation, which determines a strong interannual variability. In the warm season (from May to September), the synoptic pressure gradients are generally weak and a local, diurnal wind pattern is routinely observed. The latter is forced by surface temperature contrast generated by daytime heating and nighttime cooling and results in a southwestern breeze in the afternoon hours (characterized by an average wind speed ranging between 3 and 5 m s^−1^) and in very light or calm winds during the night and in the early morning. The absence of ventilation during the nocturnal hours is linked to the temperature inversion, a meteorological phenomenon very common in the Sabato Valley, which forms due to the cooling on the ground due to long-wave radiation in absence of synoptic scale forcing and relevant cloud cover. It is important highlighting that during winter, the temperature inversion in the Sabato Valley can last for a few days, being triggered and sustained by anticyclonic conditions at synoptic scale, as opposed to the nighttime summer season inversions, which are destroyed each day by convective motions occurring in the surface layer [27].

### 2.2. Data Sources

The air quality measurements involved in this study were data collected by ARPAC and AURA network. The ARPAC stations monitor the PM concentrations (PM_2.5_ and PM_10_), ozone (O_3_), carbon monoxide (CO), benzene (C_6_H_6_) and nitrogen dioxide (NO_2_). More specifically, PM_10_ and PM_2.5_ are routinely sampled using an automatic gravimetric instrument (OPSIS AB, model SM200), CO is measured using an infrared based instrument (Teledyne API, model T300), O_3_ is determined using a photometric based analyzer (Teledyne API, model T400), benzene is measured using a gas chromatograph (GC955, SYNSPEC BV), and NO_2_ is determined using a chemiluminescence analyzer (Thermo Fisher Scientific, model 42i). The two reference ARPAC stations are in the urban center of Avellino city and are about 1 km away from each other. The monitoring station Avellino AV41 Sc. V Circolo, (40°55′07.4″ N, 14°47′07.7″ E), hereafter AV1, monitors the levels of NO_2_, O_3_, PM_10_ and PM_2.5_. The second station, Avellino Scuola Alighieri (40°55′23.1″ N, 14°47′12.4″ E), hereinafter defined as AV2, is a traffic monitoring station and measures the levels of NO_2_, CO, C_6_H_6_, PM_10_ and PM_2.5_. The hourly values were averaged for each day to obtain daily average concentrations for each pollutant. The position of these two stations is highlighted in Figure 1 by yellow circles. To better characterize the pollution events that occurred in Avellino during September 2021 and to comprehensively describe the relationship with meteorological conditions, we have included in our dataset eight low-cost air quality stations belonging to the AURA network, Atripalda (hereafter AT), San Michele di Serino (SMS), Avellino Via De Conciliis (AVC), Torrette di Mercogliano (TM), Mercogliano (ME), Aiello del Sabato (AdS), Summonte (SU) and Monte Partenio (MP). In addition, the AURA network also includes Montevergine Observatory (MO) station, which is equipped only with meteorological instruments [28]. The location of these stations are marked as filled-in blue circles in Figure 1. As per Table 1, which lists the AURA stations involved in our work showing their geographical coordinates, the AURA monitoring devices are located at different altitudes and therefore provide very useful information about the vertical profile of PM within the PBL. We integrated the AURA dataset with the meteorological data collected by five automatic weather stations (AWS), managed by the nonprofit organization MVOBSV—Mount Vergine Observatory with the support of the University of Naples Parthenope [29]. As revealed by Table 1, four of these AWS are colocated with air quality PMSA003 devices (AVC, ME, SU and MP). The AWS include several instruments, i.e., a temperature sensor (platinum wire thermistor) and a humidity sensor (a film capacitor element), both placed in a passive radiation shield, a rain gauge, an anemometer, a pyranometer and a barometer, which perform near-continuous measurements. The meteorological and air quality data collected by the AURA network are stored on a dedicated server with a temporal resolution of 10 min. Table S1 (see Supporting Material) lists the specifications of meteorological instruments currently operating in the study area.

The AURA low-cost devices, named Airlink, are manufactured by Davis Instruments, US, and are equipped with Plantower PMSA003 sensors. The latter is able to detect the concentration of the PM_10_, PM_2.5_ and PM_1_ every minute using the principle of laser scattering. The performance of Plantower PMSA003 sensors has been evaluated against reference sensors in several previous studies [30,31,32,33,34]. Such works gave encouraging evidence about the reliability of this sensor compared to customary instruments, as testified by the very good results obtained in terms of Pearson′s linear correlation coefficient and coefficient of determination. However, in several field and laboratory experiments carred out in [30], it was found that the accuracy of PMSA003 sensor decreases when relative humidity is greater than 50%.

## 3. Results and Discussion

### 3.1. PM_2.5_ and PM_10_

For a preliminary evaluation of AURA sensor performance, we compared the PM_10_ measures collected AVC station with the data recorded by the AV1 reference station, managed by ARPAC. Among the two reference monitoring stations, AV1 and AV2, we chose AV1 because it uses the principle of laser scattering (the same as low-cost sensors). The comparison was performed on an hourly basis for the period 1 to 30 September 2021. In order to take into account the effect of hygrometric conditions, we partitioned the available data in different relative humidity (RH) classes (RH < 50%;50% ≤ RH < 60%; 60% ≤ RH < 70%; 70% ≤ RH < 80%; RH ≥ 80%). The RH data were retrieved from the AWS colocated with the AVC sensor. To synthetize the results of our analysis, we used a metric based on two simple and popular scores: the coefficient of correlation (⍴) and the BIAS (see [33]). The results, shown in Table S3 of the Supporting Material, are generally in agreement with previous studies and indicate that low-cost sensor performance is strongly dependent on hygrometric conditions. For RH < 70%, there is a general underestimation of PM_10_ values compared to reference sensors, whereas for RH greater than 70%, the BIAS changes its sign (i.e., the AURA station overestimates the PM_10_ concentration). For very high RH values (>80%), the BIAS is 32.4%. The linear correlation between the two stations is generally good and maximizes for 60% ≤ RH < 70%.

Moreover, as a further term of comparison, we evaluated the linear correlation between daily PM_10_ values measured by AV1 and AVC stations. We observed a strong association (ρ = 0.82) for data collected in September 2021, as demonstrated by Figure S12 in the Supporting material.

It should be kept in mind that our comparison is based on a couple of sensors that are not located in the same place (i.e., the distance between AV1 and AVC station is 1.1 km). Therefore, in some circumstances the difference in PM_10_ values may be dependent on local pollution sources. Moreover, our analysis is based on a data sample that covers only one month; although during September 2021 variable PM and atmospheric conditions have been observed, as it will be demonstrated in the Section 3, such data sample cannot be considered exhaustive of all possible pollution and meteorological scenarios. Therefore, in light of such considerations, we leave for future work an in-depth comparison between AURA and reference air quality sensors.

**Figure 2:**
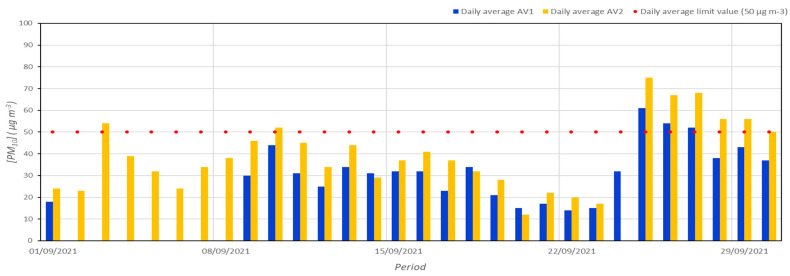
Daily average concentrations of PM_10_ during September 2021 at AV1 and AV2 monitoring stations.

The PM_10_ concentrations for the two reference monitoring stations are sketched in Figure 2. During September 2021, 3 and 7 exceedances of the limit value of 50 µg m^−3^ has been recorded for AV1 and AV2 stations, respectively. These results are in line with those obtained in a recent work about the air quality evaluation in Avellino during September 2020, when four exceedances for AV1 and 9 for AV2 were detected [26]. The highest values of 61 µg m^−3^ (at AV1) and 75 µg m^−3^ (at AV2) were measured on 25 September, in concomitance with very stable atmospheric conditions that inhibited pollutant dispersion (see also Section 3.3). For both the fixed monitoring stations, the PM_10_ average monthly concentrations in Avellino (31.9 µg m^−3^ for AV1 and 39.2 µg m^−3^ for AV2) are higher than the World Health Organization (WHO) recommended maximum value of 15 µg m^−3^ (annual mean) for the protection of public health [23].

**Figure 3:**
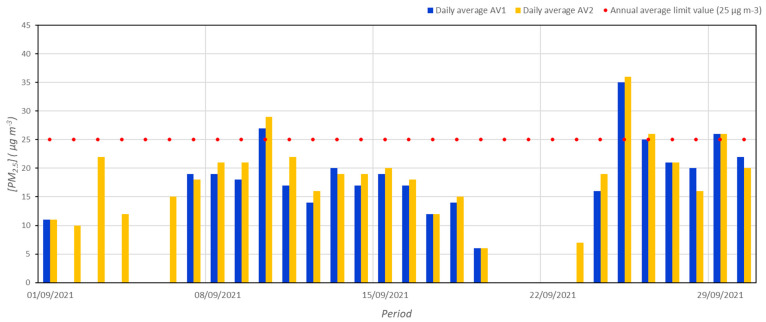
Daily average concentrations of PM_2.5_ during September 2021 at AV1 and AV2 monitoring stations.

As regards the concentrations of PM_2.5_, as revealed by Figure 3, both AV1 (18.8 µg m^−3^) and AV2 (18.3 µg m^−3^) stations exceed the recommended limit of 5 µg m^−3^ defined by the WHO for the protection of public health [23]. The highest values were measured on September 25 (35 µg m^−3^ for AV1 and 36 µg m^−3^ for AV2) by analogy with PM_10_. The trend of concentrations is very similar to that of PM_10_ as highlighted by Person’s correlations (see Table 2).

It is reasonable to assume that biomass burning in Avellino and neighboring areas exerted a relevant role on PM exceedances observed during the investigated period. Generally, during the summer season (i.e., from June to August) the biomass burning was not allowed due to the risk of fire and for this reason September sees mainly continuous agricultural activities, which are also characterized by biomass burning as final treatment of the production processes. Furthermore, to date, no policies able to eliminate the biomass burning phenomena were put in place excluding municipal ordinances for the prohibition of burning (in the Campania region, the ban on biomass burning was enacted on 15 June and was valid to 20 September, while in Avellino an additional municipal directive was enacted on 28 September 2021). Furthermore, both August 2021 (one exceedance for AV1 and three exceedances for AV2) and October 2021 (two exceedances for AV1 and three exceedances for AV2) had a lower number of cases compared to September 2021 (three exceedances for AV1 and seven exceedances for AV2). Comparable results were obtained in 2020 (August one exceedance for AV1 and one exceedance for AV2; September four exceedances for AV1 and nine exceedances for AV2; October two exceedances for AV1 and six exceedances for AV2) and in 2019 (August no exceedances for AV1 or AV2; September no exceedances for AV1 and two exceedances for AV2; October no exceedances for AV1 or AV2). These results justify the choice of September as the period most affected by agricultural waste burning in the countryside, as subsequently highlighted by AURA network data.

### 3.2. Other Relevant Atmospheric Pollutants and Pearson Correlations

The concentrations of NO_2_ for both the monitoring stations do not exceed the legislative limit of 200 μg m^−3^ as maximum hourly value with mean values of 46 μg m^−3^ for AV1 and 35 μg m^−3^ for AV2 (see Supporting Material). These results are in line with those obtained during September 2020 (38 μg m^−3^ for AV1 and 44 μg m^−3^ for AV2) due to the light traffic in Avellino. Even C_6_H_6_, the CO and O_3_ atmospheric concentrations do not exceed the corresponding legislative limits (see Supporting Material). Pearson’s correlation coefficient (ρ) is reported for PM_10_, PM_2.5_ and NO_2_, which are measured by both the monitoring stations. Table 2 reveals relevant positive correlations between the investigated pollutants. PM_10_ and PM_2.5_ for AV1 and AV2 show very strong associations between the two monitoring stations, which are highly correlated. These results could be associated with similar PM (PM_10_ and PM_2.5_) sources for both stations. For specific correlations of NO_2_ with the PM_10_ and PM_2.5_, the results reveal strong associations and are in line with data reported in [35] (ρ = 0.7 for NO_2_/PM_2_._5_). Furthermore, the lower correlations between NO_2_ and PM_10_/PM_2.5_ highlight the role of different sources for those pollutants.

In addition, data concerning the two reference monitoring stations of Avellino are discussed together with those obtained with the AURA network (AdS; SMS) located in the rural area bordering Avellino (see Figure 1). This approach allows the study of PM distribution within an area that embraces the entire Sabato Valley. The abovementioned areas are characterized by intensive agricultural activities that represent the predominant economic activity, whereas traffic and industrial activities can be considered absent or scarcely significant as PM sources.

**Figure 4:**
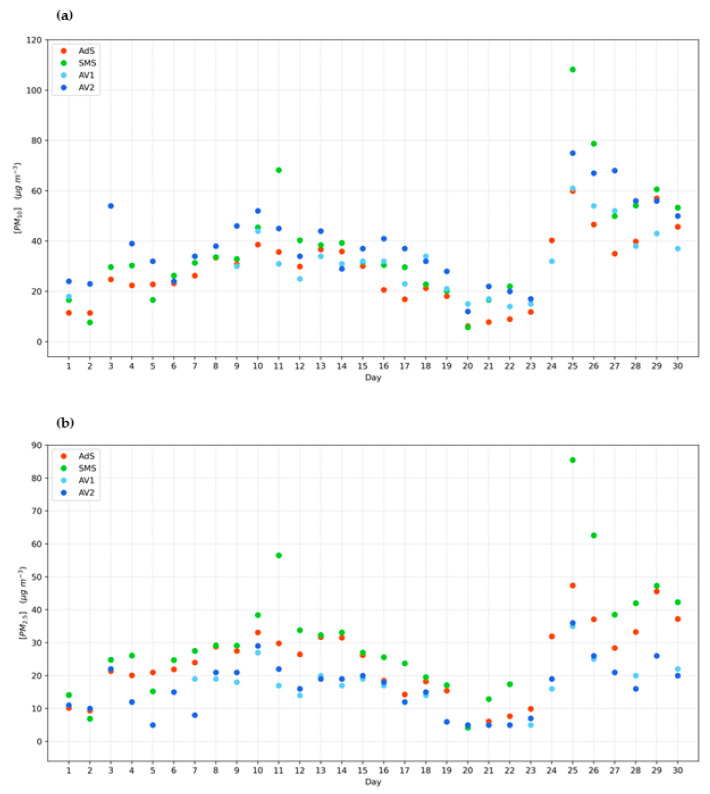
Daily average concentrations of PM_10_ (**a**) and PM_2.5_ (**b**) during September 2021 at AV1, AV2 and AURA (AdS; SMS) monitoring stations.

Results reported in Figure 4 show exceedances of the monthly average values for PM_10_ (AdS: 28.3 μg m^−3^; SMS: 37.2 μg m^−3^) for all the rural monitoring stations in concomitance with the registered exceedances in Avellino (excluding 3 September 2021). These results represent a clear indication of an extended area characterized by critical and similar conditions that define very strong associations for PM_10_ and PM_2.5_ among the investigated monitoring stations, as reported in Table 3.

**Figure 5:**
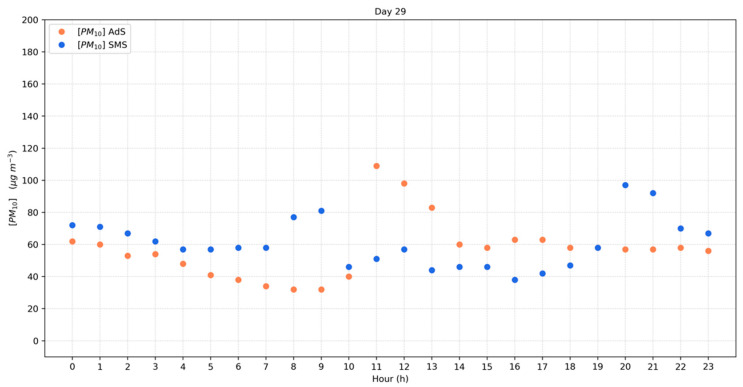
Hourly average concentrations of PM_10_ during 29 September 2021 at two AURA rural monitoring stations (AdS: Aiello del Sabato; SMS: San Michele di Serino).

Different PM sources characterize Avellino and the neighboring areas, but clear evidence on the impact of agricultural waste burning on PM atmospheric concentrations is herein reported. A useful insight on the impact of agricultural waste burning is shown in Figure 5, which presents the PM_10_ hourly average observed in AdS and SMS stations on 29 September. A close inspection of this figure reveals that the higher PM values were measured during the early hours of the day (109 μg m^−3^ at 11:00 a.m. for AdS and 81 μg m^−3^ at 9:00 a.m. for SMS), in concomitance with biomass burning process, and during the night due to thermal inversion phenomena.

### 3.3. Meteorological Conditions Observed in September 2021

In this section, we carry out a detailed analysis of the atmospheric conditions observed in the investigated period (1–30 September 2021). Following the previous literature (e.g., [19,20]), we have firstly considered two parameters that effectively represent large-scale circulation impact on local air quality levels, the 500-hPa geopotential height (Z500) and the sea-level pressure (SLP). Both fields have been retrieved from the ERA5 reanalysis dataset (https://cds.climate.copernicus.eu/cdsapp#!/search?type=dataset, last access on 23 August 2022). The comparison between synoptic parameters and PM_10_ levels observed in Avellino has been carried out on daily basis using the following approach: for Z500, we have selected the ERA5 grid point (41.0° N, 14.75° E) closest to Avellino city, in order to take into account the most representative value for the study area; for SLP, we have considered the gradient of isobaric surfaces, which is indicative of low-level synoptic flow intensity and direction. More specifically, for the purposes of this study, we defined the sea level pressure gradient (ΔP) as the difference (in hPa) between the SLP observed in the grid point nearest to Torino (46.25° N, 9.0° E), located in northwestern Italy, and the SLP observed in the grid point closest to Otranto (40.0° N, 18.5° E), located in southeastern Italy. These two points are about 1000 km from each other and therefore they provide a very good measure of pressure gradients forcing over the study area.

Moreover, we have analyzed the relationship between local meteorological conditions and PM_10_ levels through (i) the daily average wind speed (WS) observed by the Automatic Weather Station (AWS) operating in Avellino city (AVC), and (ii) the local vertical thermal gradient, defined as the ratio (ΔT/ΔZ) of the difference in minimum daily temperature between two stations relative to their difference in altitude. The ΔT/ΔZ parameter is a valid indicator of atmospheric stability (e.g., [36]), especially when inversion occurs. We have computed this indicator using two pairs of stations, SU and AVC, hereafter (ΔT/ΔZ)_1_, and MO and SU, hereafter (ΔT/ΔZ)_2_. These three stations are located at very different altitudes (see Table 1 and Figure 1), and therefore allow a proper characterization of vertical thermal gradients within the planetary boundary layer (PBL).

**Figure 6:**
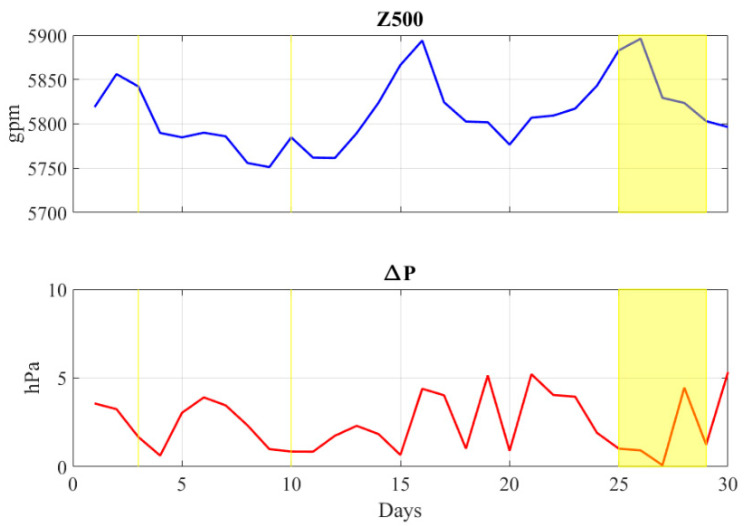
Daily 500 hPa geopotential height (blue line) and sea-level pressure gradient (red line) observed in the study area during 1–30 September period. Both fields, expressed in gpm and hPa, respectively, have been retrieved from ERA5 reanalysis. The yellow-shaded regions indicate the periods in which at least one of the reference air quality stations recorded an exceedance of daily PM_10_ levels.

Figure 6 shows the behavior in time of Z500 and ΔP parameters. The yellow-shaded regions indicate the periods in which at least one of the reference air quality sensors (managed by ARPAC) recorded a PM_10_ daily average value above the legislative limits. In the investigated period, the mid-troposphere circulation was modulated by a subtropical ridge in three periods: 1–3 September, 14–16 September and 24–27 September. In these intervals, Z500 reached values greater than 5850 gpm, which can be considered synonymous with strong subsiding motion and very light vertical thermal gradients. It should be highlighted that five of the seven PM_10_ exceedances were recorded in two of these periods, more specifically on days 3, 25, 26, 27 and 28. In almost all cases, ΔP values between 0.0 and 1.7 hPa have been detected. The only exception is day 28, when ΔP = 4.5 hPa; however, it should be noted that on this day, only the AV2 station recorded a PM_10_ daily value above 50 μg m^−3^.

As regards the other two PM_10_ exceedances (day 10 and day 29), they occurred in a large-scale atmospheric scenario characterized by a weak upper-level cyclonic area and by very low ΔP values (0.9 and 1.2 hPa, respectively). It is worth noting that in the period 14–16 September, no violations of PM_10_ daily threshold were observed: in this interval, only on day 15 (in which Z500 = 5866 gpm and ΔP = 0.7 hPa) there was a large-scale atmospheric set-up clearly favorable to air stagnation at low atmospheric levels. It can be argued that on this day, the emissions from agricultural burning were too low to produce high PM_10_ levels.

**Figure 7:**
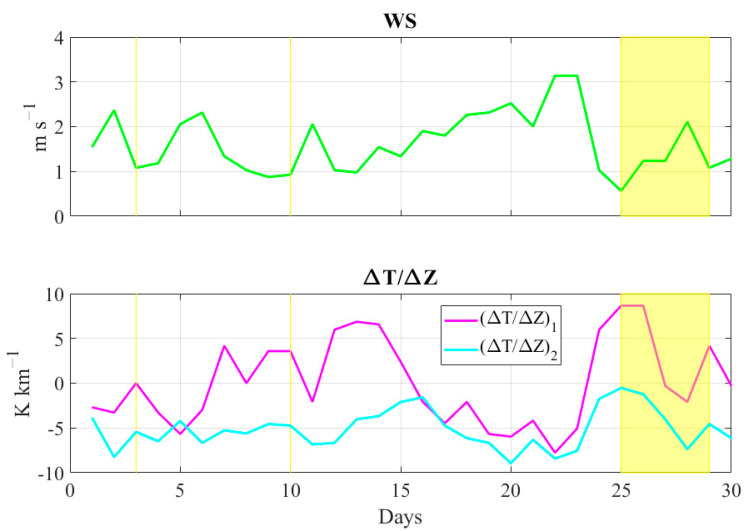
Daily average wind speed (green line) and vertical thermal gradients (magenta and cyan lines) observed in the study area during 1–30 September period. Both fields, expressed in m s^−1^ and K km^−1^, respectively, have been retrieved from meteorological weather stations belonging to the AURA network. The yellow-shaded regions indicate the periods in which at least one of the reference air quality stations recorded an exceedance of daily PM_10_ levels.

The local meteorological parameters (Figure 7) well reflect the large-scale imprinting. On days when PM_10_ values were above the limits, WS was generally near or below 1.0 m s^−1^ and (ΔT/ΔZ)_1_ was generally positive or neutral, indicating temperature inversions (the only exception is day 28, when WS = 2.1 m s^−1^ and (ΔT/ΔZ)_1_ = −2.1 K km^−1^). The (ΔT/ΔZ)_2_ indicator shows a relevant reduction in periods 14–16 and 25–27 September, when the atmospheric circulation was conditioned by a ridge. On day 25 (the case study of our work), a very unusual and critical atmospheric scenario occurred, characterized by very light winds (WS = 0.6 m s^−1^), strong temperature inversion at low levels ((ΔT/ΔZ)_1_ = +8.7 K km^−1^) and very stable atmospheric conditions at intermediate levels ((ΔT/ΔZ)_2_ = −0.5 K km^−1^). In the next section, we analyze and discuss the meteorological and air quality conditions that occurred on this day.

### 3.4. A Focus on the Case Study of 25 September 2021

**Figure 8:**
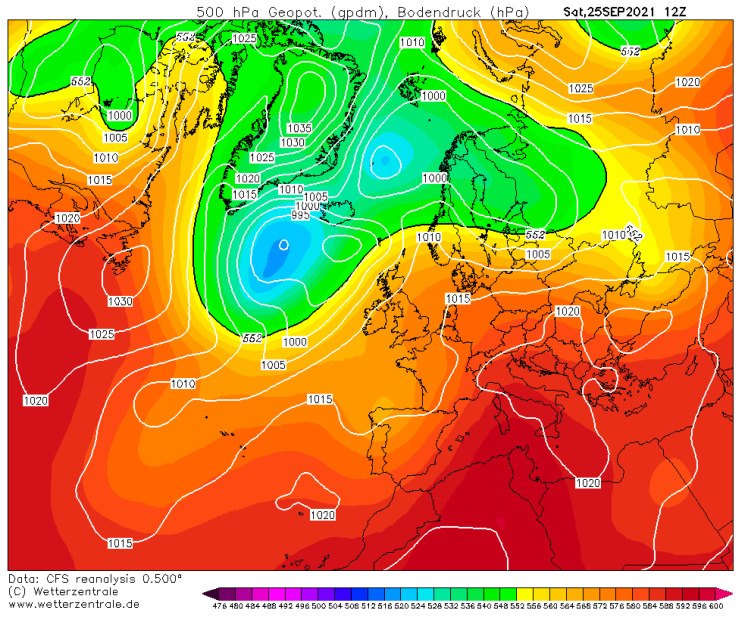
Geopotential height at 500 hPa (shaded color) and sea level pressure (white solid line) fields at 12:00 UTC of Saturday, 25 September 2021. Fields were retrieved from Climate Forecast System Reanalysis of National Centers for Environmental Prediction and are expressed in geopotential decameters and in hPa, respectively. Image is courtesy of www.wetterzentrale.de (last access on 18 March 2022).

In this paragraph, we offer a detailed description of the pollution event that occurred in the Sabato Valley on 25 September 2021. We introduce the discussion of this case study by illustrating the meteorological synoptic scenario. Figure 8 shows the Z500 and SLP fields at hour 12:00 UTC retrieved from Climate Forecast System Reanalysis of National Centers for Environmental Prediction (www.wetterzentrale.de, last access on 18 March 2022). In the Central Mediterranean area, the atmospheric conditions were modulated by a subtropical ridge, which is bounded on the western side by a stretched trough elongated towards the Iberian Peninsula. As revealed by Figure 8, the ridge was associated with very weak pressure gradients at low levels. This large-scale configuration resembles one the typical pattern that promotes the incoming, over the Central Mediterranean basin, of very warm tropical continental air mass. The latter originates from the interior of North Africa (in the Sahara region, which is notoriously a warm source region) and is usually associated with low relative humidity values. This synoptic set-up lasted three days (from 24 to 26 September) and resulted, in the study area, in strong subsiding motions (i.e., sinking motions that trap air near the surface), in very light winds, mainly forced by local diurnal regime, and in air temperature well above the average expected for this period.

According to the air quality data collected by ARPAC, the daily PM_2.5_ (35 μg m^−3^ for AV1 and 36 μg m^−3^ for AV2) and PM_10_ (61 μg m^−3^ for AV1 and 75 μg m^−3^ for AV2) average were well above the legislative limits. The high temporal resolution measurements provided by the AURA network, in combination with the available meteorological records, allows us to characterize the evolution and the vertical distribution of PM_10_ concentrations from the surface (≊300 m asl) to 1.5 km height.

**Figure 9:**
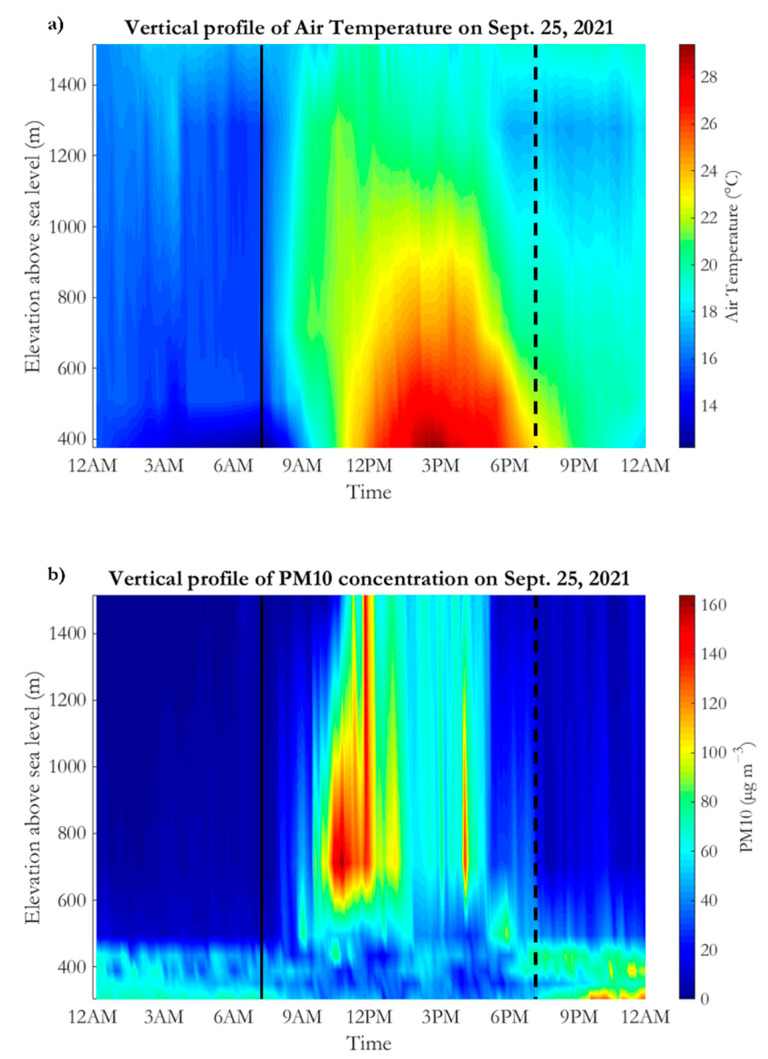
Height–time cross sections of (**a**) air temperature (in °C) and (**b**) PM_10_ concentration (in µg m^−3^) in the Sabato Valley during 25 September 2021, when a relevant pollution event occurred. The vertical profiles have been reconstructed using meteorological and air quality stations. The solid and dashed black lines on panel (**a**) indicate the time of sunrise and sunset, respectively.

Figure 9 shows the height–time section of air temperature (Figure 9a) and PM_10_ concentration (Figure 9b) during 25 September 2021. The time resolution of both air temperature and PM_10_ is 10 min. The temperature vertical profiles have been reconstructed, through a simple linear interpolation, using AVC, ME, SU, MO and MP stations, whereas PM_10_ vertical profiles were retrieved, using the same interpolation scheme, from AT, AVC, TM, ME, SU and MP air quality data. Note that the selected air quality and meteorological stations (belonging to the AURA network) are well separated in height among each other and better represent the incidence of the orography on PBL structure, especially during the night hours. It is important to highlight that, unfortunately, no other useful data sources (such as radio soundings, wind profiler and LIDAR measurements) are available in the study region.

Starting from the nocturnal hours (Figure 9a), i.e., from 0:00 to 6:00 a.m. (local time), it is very easy to recognize the presence of a well-pronounced stable boundary layer (SBL), i.e., of a statically stable layer with weak turbulence and light winds. The bottom part of the SBL, usually called the surface layer, was gradually chilled by contact with the radiatively cooled ground. The result is the formation of a surface temperature inversion, which is a typical signature of night-time PBL structure in the Sabato Valley. The surface temperature inversion reached the maximum intensity around 7:30 local time (near the sunrise), when the air temperature measured in AVC was about 3 °C lower than that recorded in ME station. Above the surface layer (which has a vertical depth of about 250 m), the air temperature rises slowly with height, reaching the highest values (≊17 °C) at the top of the analyzed atmospheric vertical slice. It is important to highlight that this PBL structure partly diverges from the typical nocturnal scenario observed in fair weather conditions, in which the SBL has a lower vertical depth (≊500 m) and gradually merges with a neutral residual layer, that can be considered the leftover part of the mixed layer of the previous day [37,38]. This difference with the customary nighttime PBL set-up may be ascribed to the synoptic forcing (anticyclonic ridge and tropical warm air advection), which causes an anomalous heating of medium-low tropospheric layers that alters the standard vertical temperature profiles. This situation appears to be very favorable to the pollutant stagnation in the surface layer, as confirmed by Figure 9b. In the night hours, in fact, the highest PM_10_ values (up to 75 µg m^−3^) were recorded near the surface between 300 and 500 m asl. Above this layer, thanks to the strong static stability, the PM_10_ concentration was relatively low.

**Figure 10:**
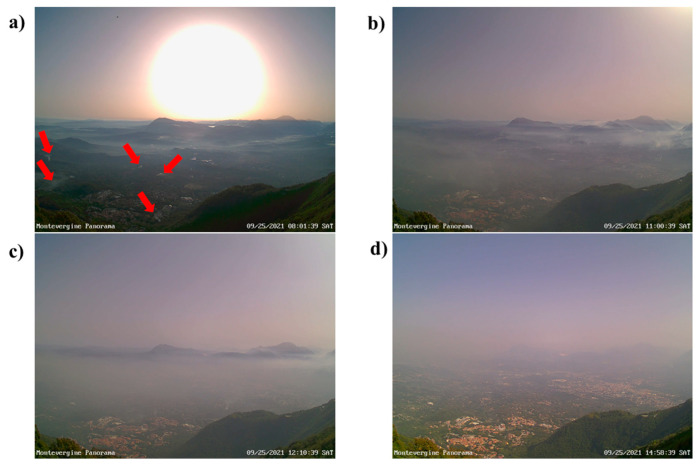
Sequence of pictures of the Sabato Valley taken at Montevergine observatory (MO) during 25 September 2021 at different hours (8:01 a.m. (**a**), 11:00 a.m. (**b**), 12:10 p.m. (**c**) and 2:58 p.m. (**d**)). The red arrows in the panel (**a**) mark some smoking areas associated with biomass burning.

After sunrise, there was rapid heating between 500 and 1300 m asl. Within this layer, the vertical temperature gradient was very weak until 11:00 a.m. and therefore very stable conditions renewed. The near-surface layer experienced a more gradual warming, which caused a partial dilution of pollutants thanks to the genesis of weak turbulent eddies. The behavior of PM_10_ concentration after 8:00 local time deserves close attention. This time, in fact, roughly coincides with the start of biomass burning activities within the Sabato Valley, mainly in countryside areas located between 350 and 800 m asl (see also Figure 5). In this respect, very useful evidence is provided by Figure 10a, which shows a picture of Sabato Valley taken from MO that clearly highlights the presence of several smoking areas caused by biomass burning (marked by the red arrows for the convenience of the reader). The PM_10_ values rapidly rose in the mid-levels of the investigated atmospheric slice, reaching a top value of 160 µg m^−3^ at the SU station around 10:50 a.m. The very pronounced atmospheric stability, as well as the very weak horizontal winds, caused a relevant and quite rare worsening of air quality between 500 and 1000 m asl (see Figure 10b). After 11:00 h, the warming of the near-surface layer intensified and the classical daytime mixed layer started to form. The latter enhanced the convective motions which transported very high pollutant amounts up to 1515 m slm, where PM_10_ values up to 148 µg m^−3^ were detected around 12:00 p.m. The vertical transport of pollutants caused by mixed layer growth can be also appreciated by comparing with Figure 10b,c, which were taken at 11:00 a.m. and 12:10 p.m., respectively. From 12:00 to 4:00 p.m., the PBL height further grew up and the turbulent mixing partly diluted the pollutant, especially between 300 and 600 m asl. The beneficial effect of turbulent mixing can be easily detected by inspecting Figure 10d, which shows an improvement of visibility from MO towards the Sabato Valley. After the sunset (around 6:00 p.m.), by virtue of a temporary increase in wind speed and the ending of biomass burning activities, there was a significant improvement of air quality above 700 m asl. Conversely, the gradual formation of a new SBL, which clearly emerges from Figure 9a after 9:00 p.m., caused an increase of PM_10_ levels at TM, AVC and AT stations. More specifically, in late evening, PM_10_ values up to 130 µg m^−3^ were recorded in the city of Atripalda.

To summarize, from the analysis of available in situ meteorological and air quality data, it emerges that the pollution event that occurred in the Sabato Valley on 25 September 2021 had a strict dependence with the synoptic and local atmospheric conditions. The strong stability observed during the nocturnal hours and after the late afternoon promoted the genesis of a surface temperature inversion that deteriorates the air quality in the city of Avellino and, more in general, in areas located below 500 m asl. Moreover, the subsidence triggered by the ridge, as well as the warm tropical air advection, altered the vertical temperature profile in the layer between 500 and 1500 m asl, causing within this, in combination with massive biomass burning emissions, a temporary but very strong pollution event in the morning hours.

## 4. Conclusions

In this work, first evidence of the effect of agricultural waste burning and meteorological conditions on air quality in Avellino and neighboring areas is discussed. The results show strong associations between the two fixed urban monitoring stations in Avellino managed by ARPAC and several rural stations, placed in the neighboring of Avellino, belonging to the AURA network. Using high-temporal resolution PM and meteorological measurements provided by AURA, we provided a detailed description of the pollution events that took place on 25 September 2021 in the Sabato Valley. Our analysis clearly demonstrated that the air quality in the investigated area is strictly dependent on both large-scale and local atmospheric conditions. In the considered case study, the rise in PM concentrations observed in Avellino city and in its neighboring areas was not only the fruit of the local nocturnal temperature inversion, but also of the strong subsidence triggered by a subtropical ridge, which limited the daytime mixing especially in the morning hours. The results of this work impose deep thoughts about the role exerted by meteorological factors on air quality dynamics observed in the Sabato Valley both from scientific and legislative perspectives. On one hand, they suggest extending the research to other relevant pollution episodes in the study area, in order to detect other synoptic types favorable to air stagnation and their variability over seasons. On the other hand, the findings of our study suggest planning weather-responsive strategies for the mitigation of PM emissions. In this respect, the introduction of a meteorological alert based on the prediction of conditions that inhibit the dispersion of atmospheric pollutants may be the base of dedicated policy decisions that could mitigate the emission of high amounts of PM in the atmosphere (i.e., stop of critical activities, alternative strategies for biomasses treatment). More specifically, in the analysis of the meteorological conditions that may trigger severe pollution events, much attention should be paid to the synoptic scenario. The latter can be efficiently synthetized by the 500-hPa geopotential height and by the sea-level pressure fields. As revealed by our analysis, it is crucial to evaluate the occurrence of a mid-tropospheric subtropical ridge, which can relax vertical temperature gradient and inhibit the vertical air mixing, as well as of weak sea-level pressure gradients, which determine very light wind preventing adequate pollution dispersion. These parameters are generally predictable with sufficient accuracy three or four days ahead. Moreover, the large-scale analysis should be associated with a careful examination of some local meteorological parameters, such as the low-level wind circulation and the vertical temperature gradient within the PBL.

The influence of the meteorological conditions on air quality in Avellino was considered and was able to define peculiar conditions that contribute to characterizing the study area like an “indoor environment” (i.e., a polluted indoor environment is characterized by the absence of ventilation, which is responsible for high pollutant concentrations). The effect of the agricultural waste burning has been investigated using the AURA network in areas bordering Avellino characterized by intense agricultural activities as a major PM source. Results show very high PM concentration (up to 195 µg m^−3^) concomitant with agricultural waste burning. The effects of those activities seem predominantly on the PM exceedances in September 2021 and have been exacerbated, in some circumstances, by very stable atmospheric conditions. The latter, as documented by our focus on the pollution event that occurred on 25 September, inhibited the pollution dispersion not only in the night hours (as usually happens in the Sabato Valley) but also during the morning.

It should be highlighted that this study has explored some of the potential advantages of a low-cost air quality device network. The latter proved to be useful for a better understanding of the linkages between pollution events and local meteorological conditions (e.g., thermal inversion, PBL structure, etc.). However, it is worth noting that low-cost devices have several shortcomings for both operational and research purposes. Their accuracy, in fact, is strictly dependent on environmental conditions, especially on relative humidity values.

Future studies based on source apportionment to quantify the contribution of different sources on PM levels seem to be necessary in order to integrate this preliminary evaluation. This approach could be useful to adopt dedicated and conscious actions to reduce the exposure to high PM concentrations. Furthermore, our research activities will be devoted to the development of a region-specific calibration model for AURA devices, which incorporates an adjustment for thermo-hygrometric conditions.

## Figures and Tables

**Table 1:** List of monitoring stations used in the study. For each site coordinates, altitude and type of station (air quality and/or weather station) are specified.

Station	Geographical Coordinates	Altitude (a.s.l.)	Air Quality Station	Weather Station
Atripalda (AT)—AURA	40°55′7.666″ N 14°49′49.332″ E	303 m	X	
San Michele di Serino (SMS)—AURA	40° 52′ 39.749″ N 14° 51′ 3.409″ E	350 m	X	
Avellino, “School Dante Alighieri,” Via Piave (AV2)—ARPAC	40° 55′ 07.4″ N 14°47′07.7″ E	356 m	X	
Avellino, “School V Circolo” Via Oscar d’Agostino (AV1)—ARPAC	40°55′23.1″ N 14°47′12.4″ E	371 m	X	
Avellino, Via De Conciliis (AVC)—AURA	40°54′52.204″ N 14°46′58.674″ E	375 m	X	X
Torrette di Mercogliano (TM)—AURA	40°54′21.816″ N 14°45′45.296″ E	388 m	X	
Aiello del Sabato (AdS)—AURA	40°54′2.509″ N 14°49′22.894″ E	390 m	X	
Mercogliano (ME)—AURA	40°55′23.102″ N 14°44′47.412″ E	495 m	X	X
Summonte (SU)—AURA	40°56′50.507″ N 14°44′35.343″ E	710 m	X	X
Montevergine Observatory (MO)—AURA	40°56′11.407″ N 14°43′44.94″ E	1280 m		X
Monte Partenio (MP)—AURA	40°56′41.074″ N 14°43′43.25″ E	1515 m	X	X

**Table 2:** Pearson correlations of NO_2_, PM_2.5_, PM_10_, for AV1 and AV2 monitoring stations during September 2021 (*p* < 0.005).

	[PM_2.5_] AV1	[PM_2.5_] AV2	[PM_10_] AV1	[PM_10_] AV2	[NO_2_] AV1	[NO_2_] AV2
[PM_2.5_] AV1	1	0.95	0.90	0.83	0.72	0.78
[PM_2.5_] AV2	0.95	1	0.87	0.81	0.82	0.79
[PM_10_] AV1	0.90	0.87	1	0.95	0.72	0.70
[PM_10_] AV2	0.83	0.81	0.95	1	0.72	0.68
[NO_2_] AV1	0.72	0.82	0.72	0.72	1	0.93
[NO_2_] AV2	0.78	0.79	0.70	0.68	0.93	1

**Table 3:** Pearson correlations of PM_2.5_ and PM_10_, for AV1, AV2 and two AURA monitoring stations (Aiello del Sabato and San Michele di Serino) during September 2021 (*p* < 0.005).

	[PM_10_] AV1	[PM_10_] AV2	[PM_10_] AdS	[PM_10_] SMS
[PM_10_] AV1	1	0.95	0.87	0.86
[PM_10_] AV2	0.95	1	0.85	0.85
[PM_10_] Ads	0.87	0.85	1	0.89
[PM_10_] SMS	0.86	0.85	0.89	1

## Data Availability

https://www.arpacampania.it (last access on 21 July 2022), http://www.mvobsv.org (last access on 20 July 2022).

## Supplementary Materials

The following supporting information can be downloaded at: https://www.mdpi.com/article/10.3390/ijerph191912246/s1.
