# Supplementary material for: The Impact of Meteorological Conditions and Agricultural Waste Burning on PM Levels: A Case Study of Avellino (Southern Italy)
Source: Int J Environ Res Public Health. 2022 Sep 27;19(19):12246. doi: 10.3390/ijerph191912246 (PMC9566629; doi:10.3390/ijerph191912246)
Supplement: Supplementary file 1 [file ijerph-19-12246-s001.zip › ijerph-1864103-supplementary.pdf]

SUPPORTING MATERIALS

The Impact of Meteorological Conditions and Agricultural Waste Burning on PM Levels: A Case Study of Avellino (Southern Italy)

Vincenzo Capozzi [1,*] , Letizia Raia [2], Viviana Cretella [1], Carmela De Vivo [1] and Raffaele Cucciniello [2,*]

[1] Department of Science and Technology, University of Naples "Parthenope,"
Centro Direzionale di Napoli—Isola C4, 80143 Naples, Italy
[2] Department of Chemistry and Biology "Adolfo Zambelli", University of Salerno, Via Giovanni Paolo II, 132,
84084 Fisciano, Italy

* Correspondence: vincenzo.capozzi@uniparthenope.it (V.C.); rcucciniello@unisa.it (R.C.)

Table S1. Technical specifications of the meteorological sensors currently operating in the study areas.

Parameter	Range	Resolution	Update Interval (s)	Uncertainty
Air temperature	-40 to +65°C	0.1°C	10 to 12	0.3°C
Relative humidity	0 to 100%	1%	50-60	2%
Atmospheric pressure	540 to 1100 hPa	0.1 hPa	60	1.0 hPa
Wind speed	0 to 89 m/s	1.0 knots	2.5 to 3	0.9 m/s
Wind direction	1 to 360 degree	1°	2.5 to 3	3°
Precipitation amount	0 to 999.8 mm	0.2 mm	20 to 24	3% for rain rate up to 250 mm/h

In table S2 are reported all the data collected in the period object of the present work, for all the investigated pollutants in AV1 and AV2 monitoring stations.

Table S2

DATE	MONITORING STATION	NO_2				CO mob			PM_{10}		$PM_{2.5}$	O_3				BENZENE		
		Hour (Maximum hourly)	Hour	Daily average	Higher hours	Hour (Maximum hourly)	Daily average	Higher hours	Daily average	Higher days	Daily average	Hour (Maximum hourly)	Hour	Daily average	Higher hours	Hour (Maximum hourly)	Hour	Daily average
01/09/2021	AV1	32	7	15	0	*	*	*	18	30	11	81	24	56	0	*	*	*
	AV2	23	7	13	0	0,5	0,5	0	24	38	11	*	*	*	*	1,6	9	0,9
02/09/2021	AV1	26	19	12	0	*	*	*	nv	30	nv	86	3	70	0	*	*	*
	AV2	20	20	9	0	0,5	nv	0	23	38	10	*	*	*	*	0,8	20	0,6
03/09/2021	AV1	50	8	22	0	*	*	*	nv	30	nv	134	17	73	0	*	*	*
	AV2	35	10	17	0	0,6	0,5	0	54	39	22	*	*	*	*	1,7	21	1,1
04/09/2021	AV1	27	9	12	0	*	*	*	nv	30	nv	91	15	46	0	*	*	*
	AV2	19	8	10	0	0,6	0,5	0	39	39	12	*	*	*	*	1,4	8	0,9
05/09/2021	AV1	18	22	9	0	*	*	*	nv	30	nv	78	16	38	0	*	*	*
	AV2	12	20	6	0	0,5	0,5	0	32	39	<5	*	*	*	*	0,7	13	0,5
06/09/2021	AV1	38	7	14	0	*	*	*	nv	30	nv	76	15	61	0	*	*	*
	AV2	19	8	11	0	0,5	0,5	0	24	39	15	*	*	*	*	0,8	10	0,6
07/09/2021	AV1	45	7	17	0	*	*	*	nv	30	19	90	15	67	0	*	*	*
	AV2	28	9	15	0	0,6	0,5	0	34	39	18	*	*	*	*	1,3	8	0,7
08/09/2021	AV1	38	8	19	0	*	*	*	nv	30	19	89	16	54	0	*	*	*
	AV2	34	10	19	0	0,7	0,6	0	38	39	21	*	*	*	*	1,8	10	1
09/09/2021	AV1	45	9	24	0	*	*	*	30	30	18	96	16	50	0	*	*	*
	AV2	31	9	20	0	0,7	0,6	0	46	39	21	*	*	*	*	1,8	9	1,2
10/09/2021	AV1	74	21	31	0	*	*	*	44	30	27	128	15	55	0	*	*	*
	AV2	50	10	26	0	0,7	0,5	0	52	40	29	*	*	*	*	2,8	9	1,5
11/09/2021	AV1	35	2	19	0	*	*	*	31	30	17	88	17	58	0	*	*	*
	AV2	26	1	19	0	0,5	0,4	0	45	40	22	*	*	*	*	2,6	1	1,3
12/09/2021	AV1	37	20	13	0	*	*	*	25	30	14	87	14	50	0	*	*	*
	AV2	36	22	16	0	0,4	0,3	0	34	40	16	*	*	*	*	1,3	24	0,8
13/09/2021	AV1	94	21	27	0	*	*	*	34	30	20	126	16	44	0	*	*	*
	AV2	48	22	25	0	0,5	0,4	0	44	40	19	*	*	*	*	2,4	11	1,3
14/09/2021	AV1	56	8	26	0	*	*	*	31	30	17	121	16	60	0	*	*	*
	AV2	43	9	21	0	0,5	0,4	0	29	40	19	*	*	*	*	2,2	9	1,1
15/09/2021	AV1	45	8	24	0	*	*	*	32	30	19	109	16	51	0	*	*	*
	AV2	41	10	22	0	0,5	0,4	0	37	40	20	*	*	*	*	2,1	9	1,2
16/09/2021	AV1	66	8	20	0	*	*	*	32	30	17	118	15	69	0	*	*	*
	AV2	49	9	19	0	0,4	0,4	0	41	40	18	*	*	*	*	1,3	9	0,9
17/09/2021	AV1	69	8	24	0	*	*	*	23	30	12	82	14	52	0	*	*	*
	AV2	54	9	22	0	0,4	0,4	0	37	40	12	*	*	*	*	1,7	9	0,7
18/09/2021	AV1	43	8	14	0	*	*	*	34	30	14	79	2	56	0	*	*	*
	AV2	36	9	13	0	0,4	0,3	0	32	40	15	*	*	*	*	1,5	9	0,6
19/09/2021	AV1	18	1	9	0	*	*	*	21	30	6	88	16	61	0	*	*	*
	AV2	18	20	9	0	0,4	0,3	0	28	40	6	*	*	*	*	0,7	1	0,4
20/09/2021	AV1	21	20	10	0	*	*	*	15	30	<5	64	15	50	0	*	*	*
	AV2	22	8	12	0	0,4	0,3	0	12	40	<5	*	*	*	*	0,6	22	0,3
21/09/2021	AV1	52	7	16	0	*	*	*	17	30	<5	76	15	50	0	*	*	*
	AV2	40	8	19	0	0,4	0,4	0	22	40	<5	*	*	*	*	2	9	0,7
22/09/2021	AV1	19	8	9	0	*	*	*	14	30	<5	83	24	66	0	*	*	*
	AV2	28	8	15	0	0,4	0,4	0	20	40	<5	*	*	*	*	1,1	10	0,6
23/09/2021	AV1	37	23	13	0	*	*	*	15	30	<5	81	1	61	0	*	*	*
	AV2	30	19	16	0	0,5	0,4	0	17	40	7	*	*	*	*	1,3	20	0,7
24/09/2021	AV1	58	23	32	0	*	*	*	32	30	16	117	17	43	0	*	*	*
	AV2	44	10	29	0	0,6	0,5	0	nv	40	19	*	*	*	*	2,1	23	1
25/09/2021	AV1	87	21	38	0	*	*	*	61	31	35	101	16	37	0	*	*	*
	AV2	67	21	35	0	0,8	0,6	0	75	41	36	*	*	*	*	3,7	22	2,2
26/09/2021	AV1	55	1	25	0	*	*	*	54	32	25	111	15	41	0	*	*	*
	AV2	40	1	23	0	0,8	0,6	0	67	42	26	*	*	*	*	3,3	9	1,9
27/09/2021	AV1	45	9	23	0	*	*	*	52	33	21	65	16	29	0	*	*	*
	AV2	38	10	21	0	0,6	0,5	0	68	43	21	*	*	*	*	2,4	10	1,3
28/09/2021	AV1	31	7	14	0	*	*	*	38	33	20	68	21	46	0	*	*	*
	AV2	25	20	16	0	0,6	0,5	0	56	44	16	*	*	*	*	2,1	3	1,1
29/09/2021	AV1	51	9	25	0	*	*	*	43	33	26	92	16	42	0	*	*	*
	AV2	43	11	25	0	0,7	0,6	0	56	45	26	*	*	*	*	3,2	9	1,5
30/09/2021	AV1	55	9	22	0	*	*	*	37	33	22	75	14	45	0	*	*	*
	AV2	39	10	25	0	0,6	0,6	0	50	45	20	*	*	*	*	2,6	9	1,5

Legend:

*: analyzer not provided for by DGRC 683/2014
m: analyzer in maintenance
nv: data that cannot be validated
np: data not received
°: station with additional analyzers compared to DGRC 683/2014
* °: station managed by CALENIA (Article 5 of Legislative Decree 155/2010)

Table S3 shows a comparison between the PM_{10} measures collected by Avellino, Via De Conciliis (AVC) station, which belongs to AURA network, with the data recorded by the Avellino, School V Circolo (AV1) reference station, managed by local environmental institution. The comparison has been performed on hourly basis for the period 01 to 30 September 2021. In order to take into account the effect of hygrometric conditions, we have partitioned the available data in different relative humidity (hereafter, RH) classes (RH < 50%; 50%≤RH<60%; 60%≤RH<70%; 70%≤RH<80%; RH≥80%). The RH data have been retrieved from the Automatic Weather Station colocated with AVC air quality sensor.

Table S3. Comparison, on hourly basis, between PM_{10} data measured by reference AV1 station (ARPAC network) and low-cost AVC device (AURA network). For different relative humidity categories, the number of available samples, the average PM_{10} value (μg m^{-3}), the Bias score (%) and correlation coefficient are shown. Data collected from 01 to 30 September have been considered.

**Relative humidity class**	**Number of samples**	**$(PM_{10})_{AV1}$ (µg m^{-3})**	**$(PM_{10})_{AVC}$ (µg m^{-3})**	**BIAS (%)**	**ρ**
RH < 50%	122	28.7	20.0	-30.5	0.58
50%≤RH<60%	108	29.3	22.6	-22.9	0.60
60%≤RH<70%	144	32.7	27.6	-15.5	0.72
70%≤RH<80%	151	33.0	35.2	6.5	0.67
RH≥80%	194	31.1	41.2	32.4	0.64

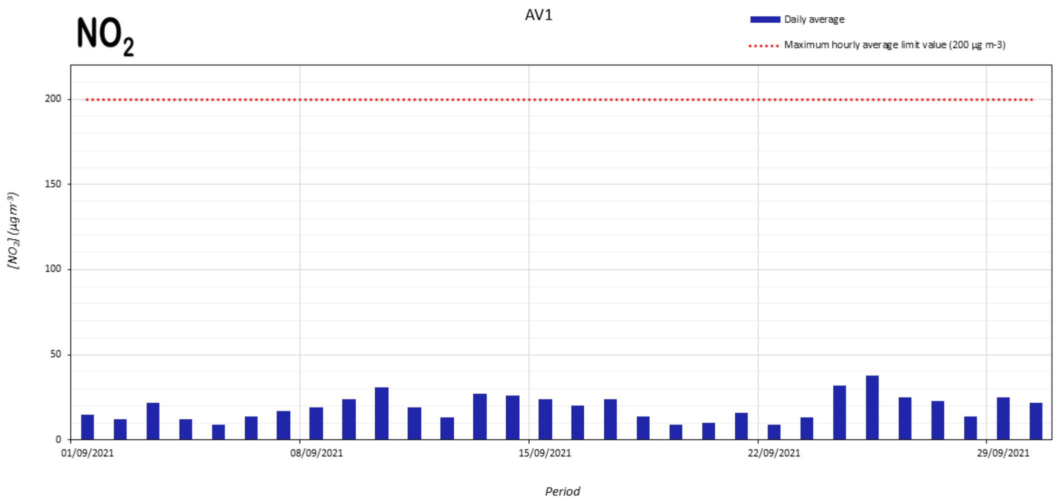

Figure S1. Daily average concentrations of NO_2 during September 2021 at AV1 monitoring station.

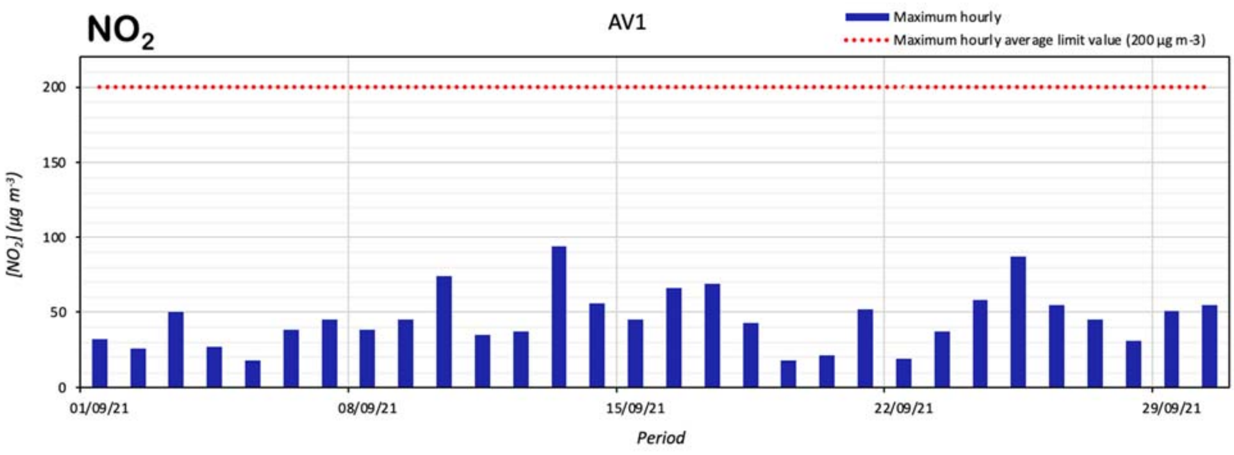

Figure S2. Maximum hourly values concentrations of NO_2 during September 2021 at AV1 monitoring station.

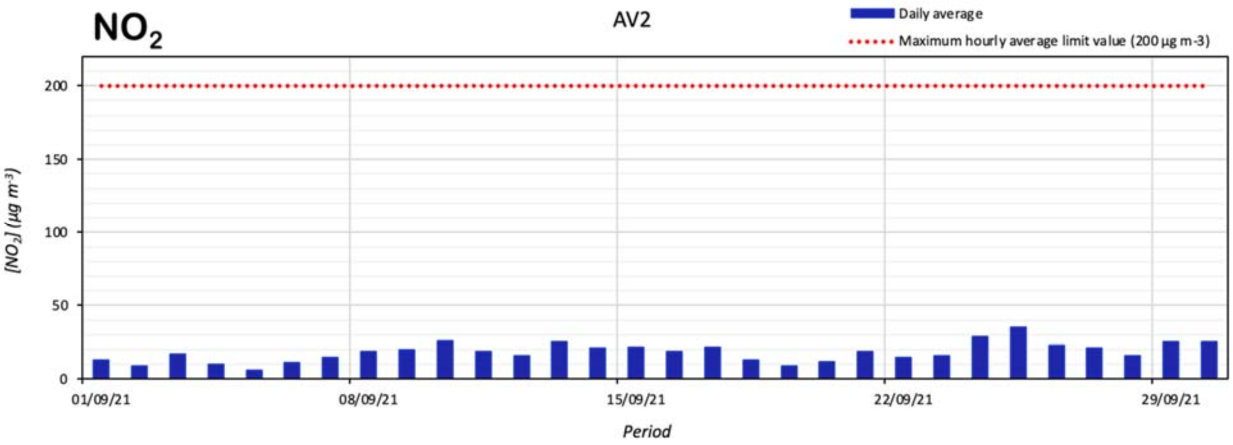

Figure S3. Daily average concentrations of NO_2 during September 2021 at AV2 monitoring station.

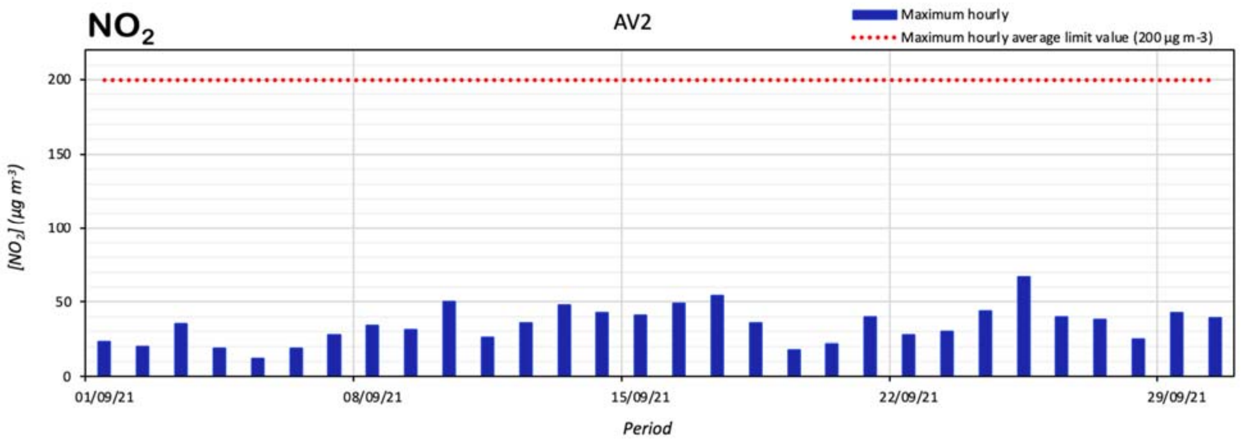

Figure S4. Maximum hourly values concentrations of NO_2 during September 2021 at AV2 monitoring station.

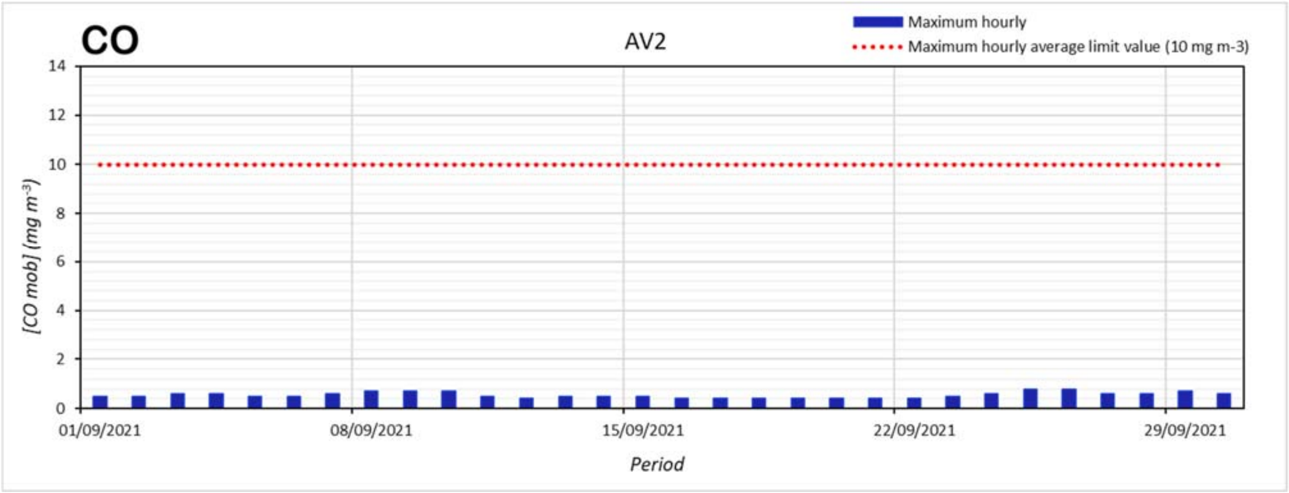

Figure S5. Maximum hourly values concentrations of CO during September 2021at AV2 monitoring station.

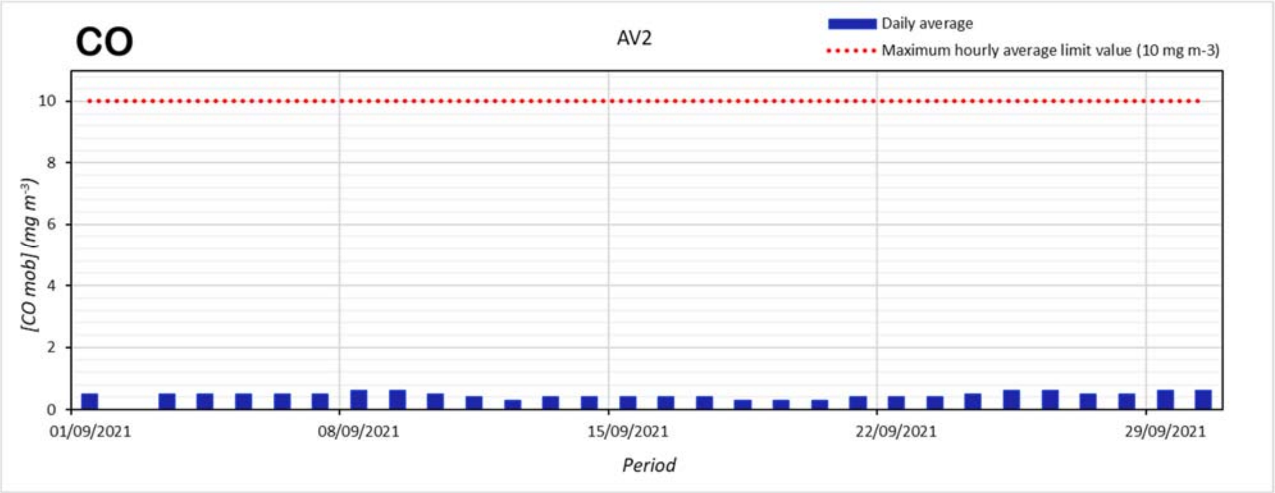

Figure S6. Daily average values concentrations of CO during September 2021at AV2 monitoring station.

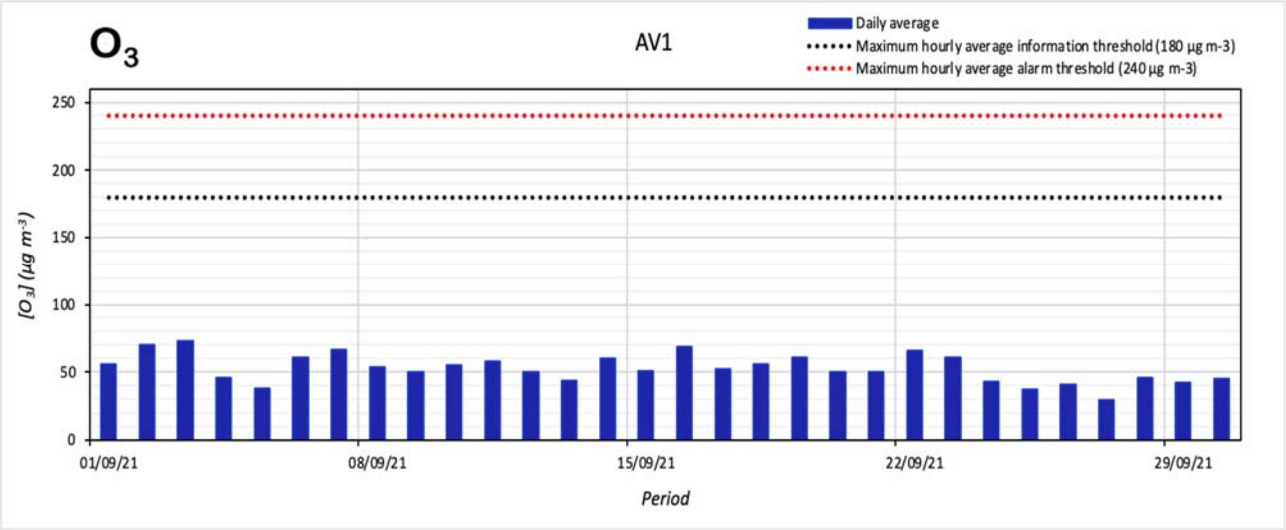

Figure S7. Daily average concentrations of O_3 during September 2021at AV1 monitoring station.

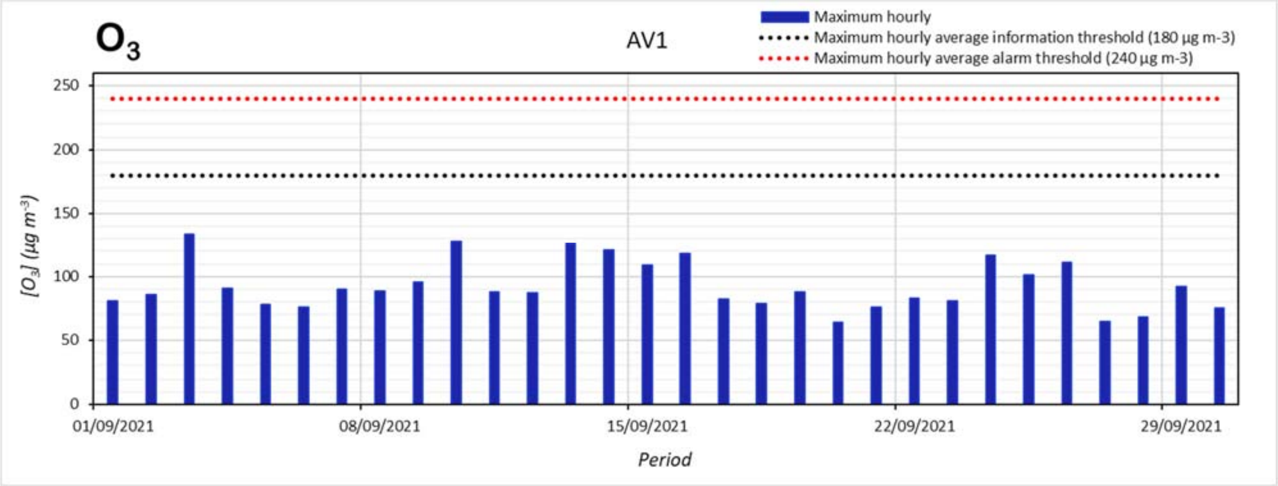

Figure S8. Maximum hourly values concentrations of O_3 during September 2021at AV1 monitoring station.

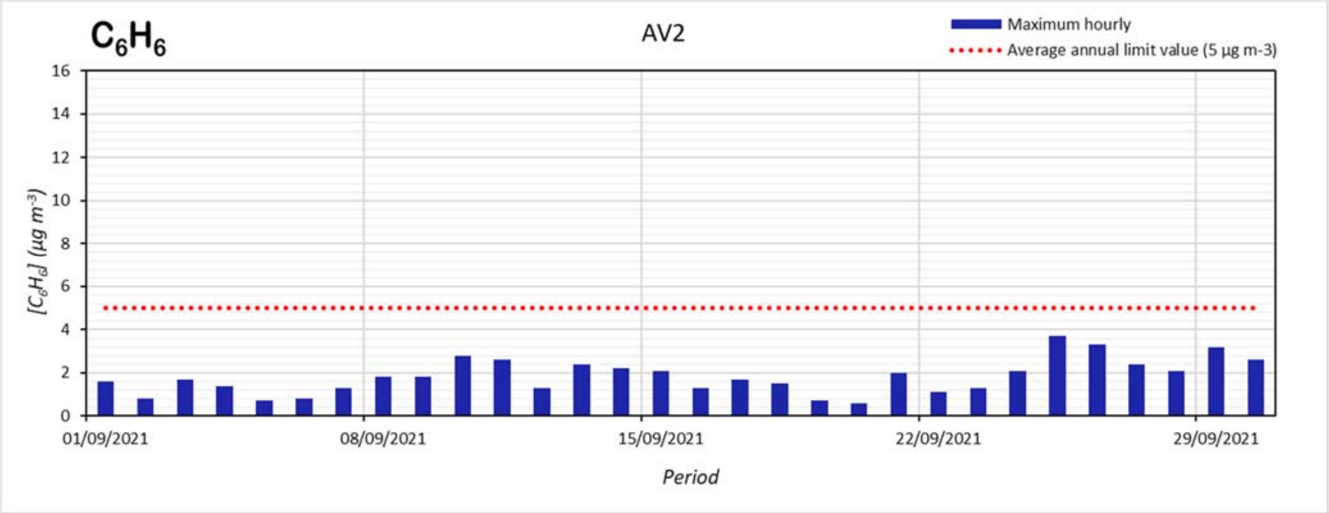

Figure S9. Maximum hourly values concentrations of benzene during September 2021at AV2 monitoring station.

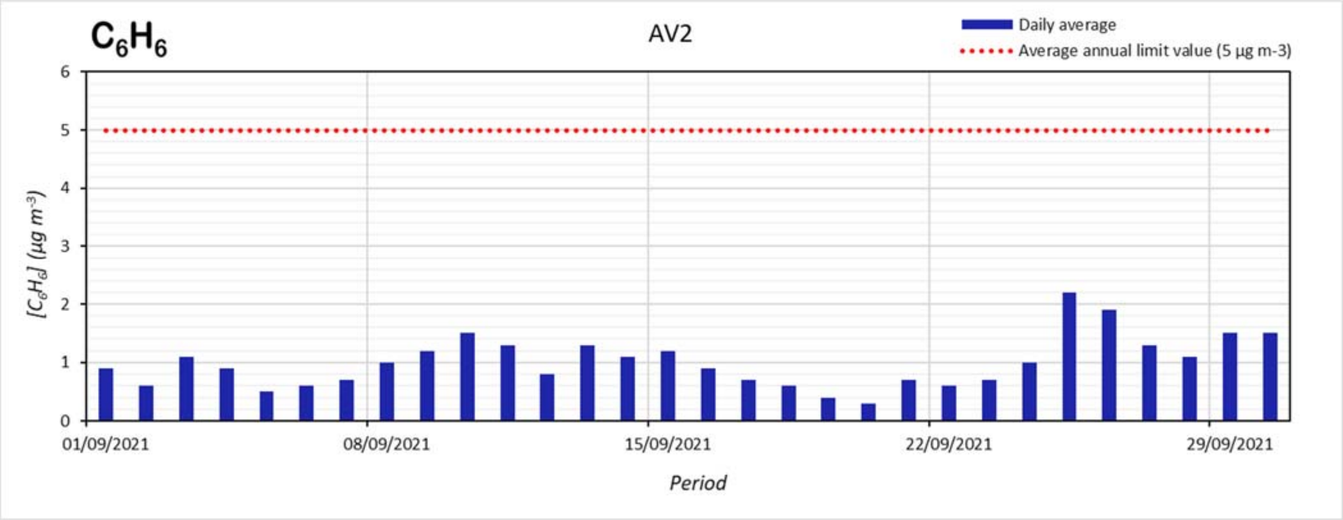

Figure S10. Daily average concentrations of benzene during September 2021at AV2 monitoring station.

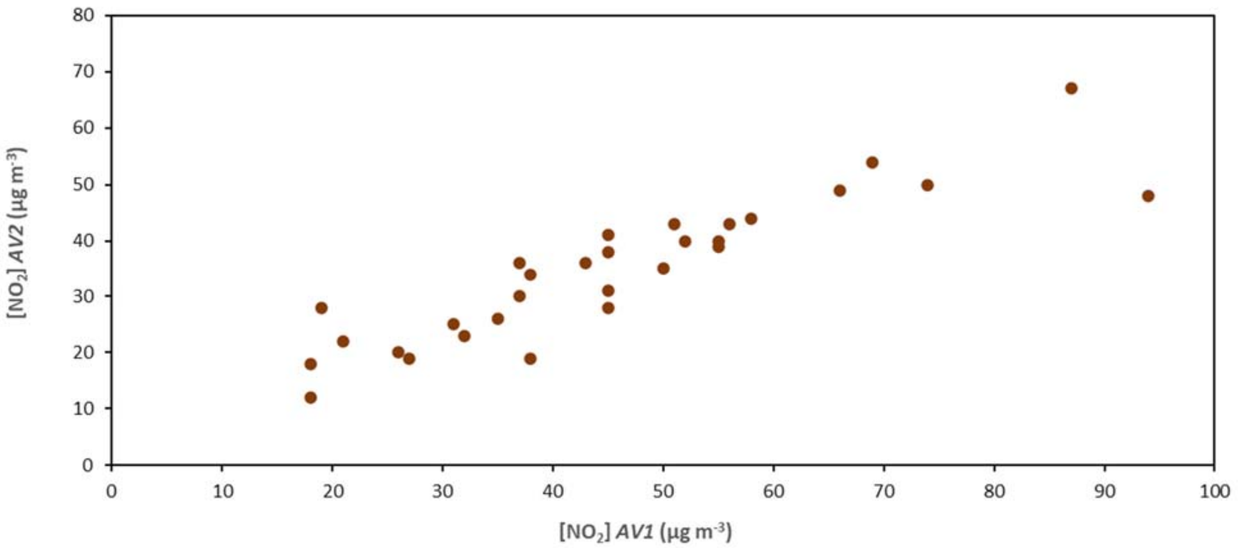

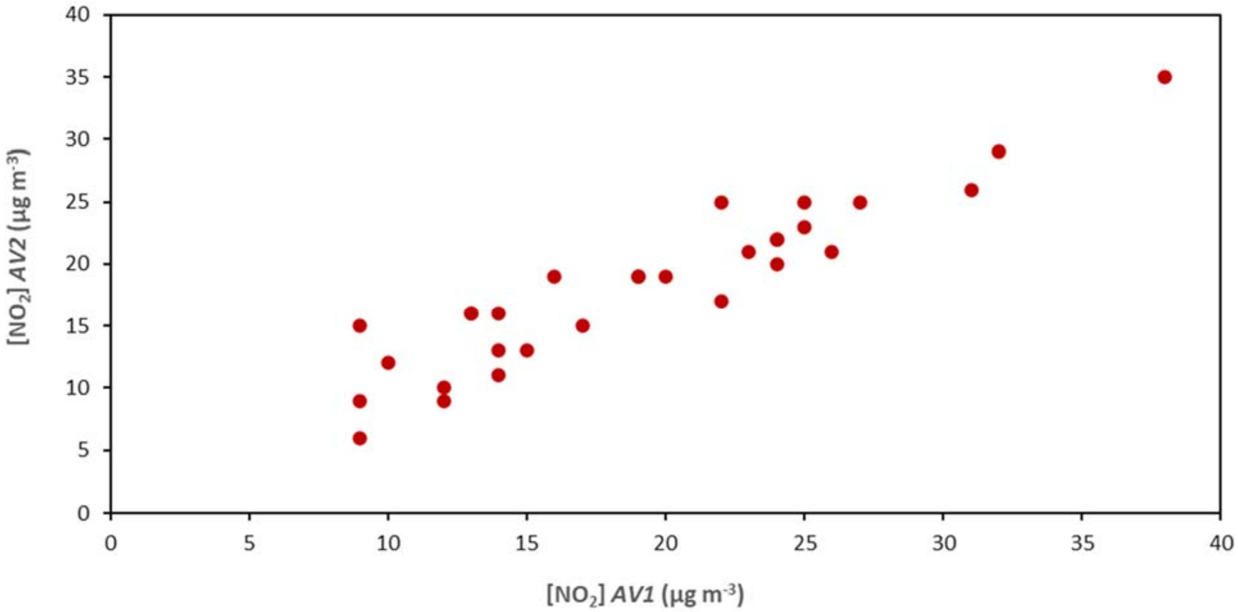

[NO2] AV2 (μg m-3)
[NO2] AV1 (μg m-3)
0
5
10
15
20
25
30
35
40

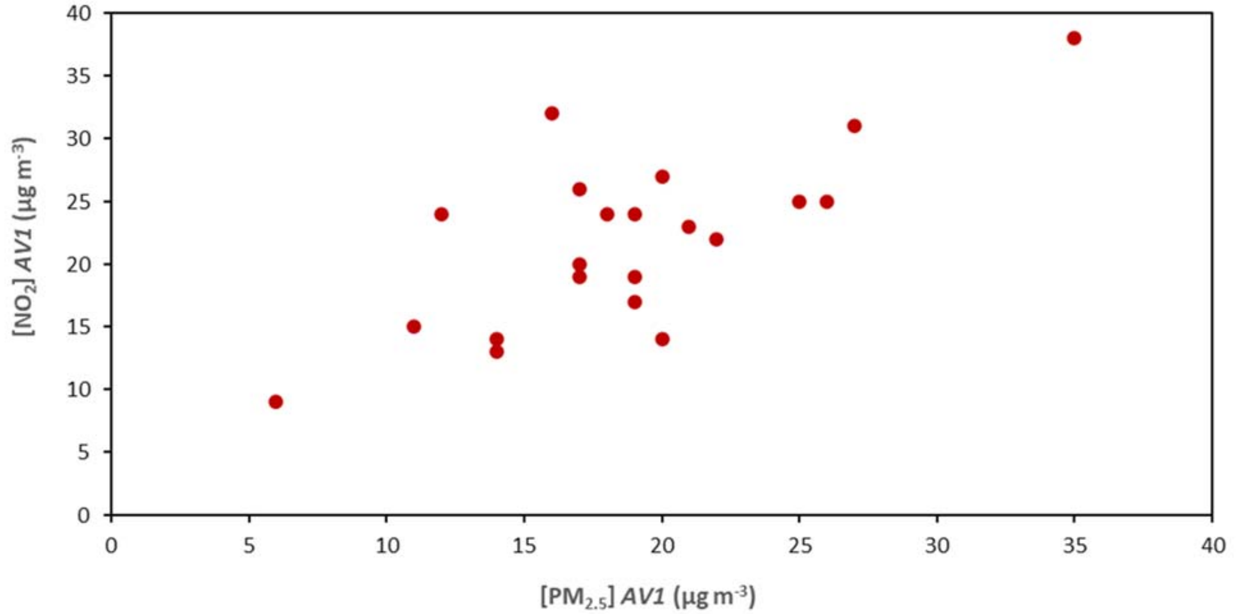

[NO2] AV1 (μg m-3)
[PM2.5] AV1 (μg m-3)
0
5
10
15
20
25
30
35
40

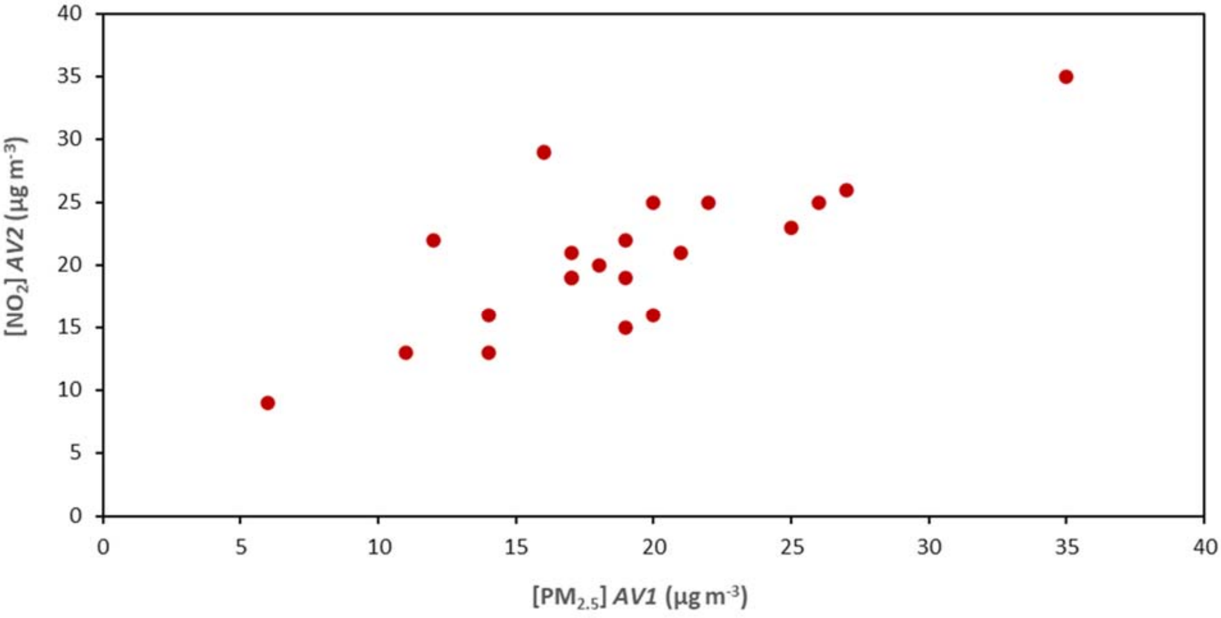
[NO2] AV2 (µg m-3)
[PM2.5] AV1 (µg m-3)
0
5
10
15
20
25
30
35
40

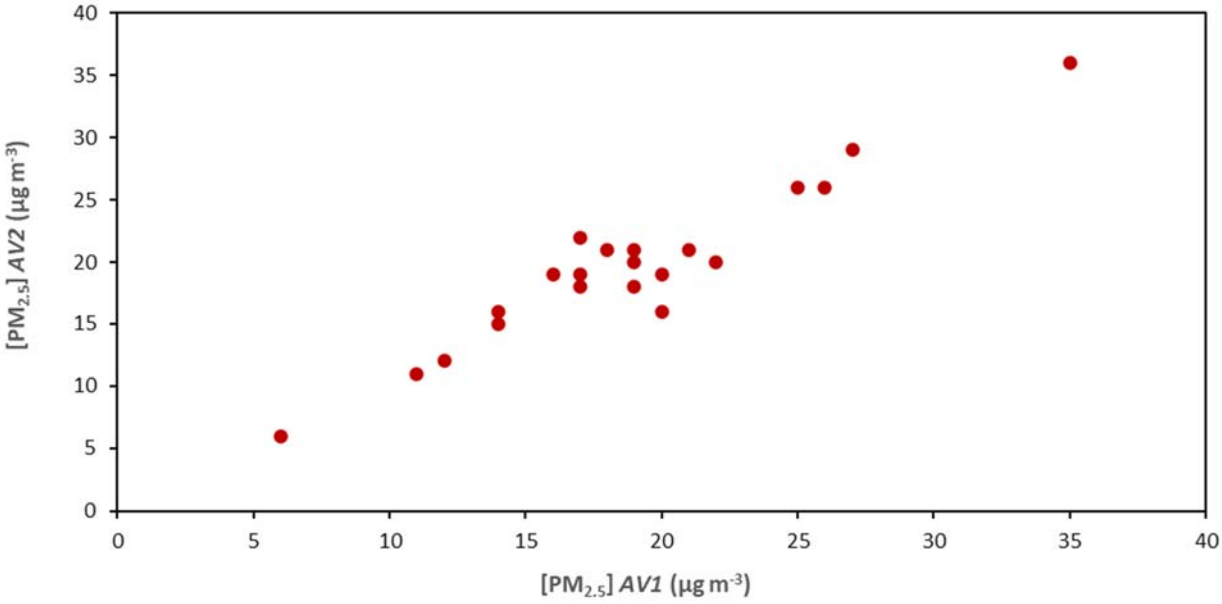
[PM2.5] AV2 (µg m-3)
[PM2.5] AV1 (µg m-3)
0
5
10
15
20
25
30
35
40

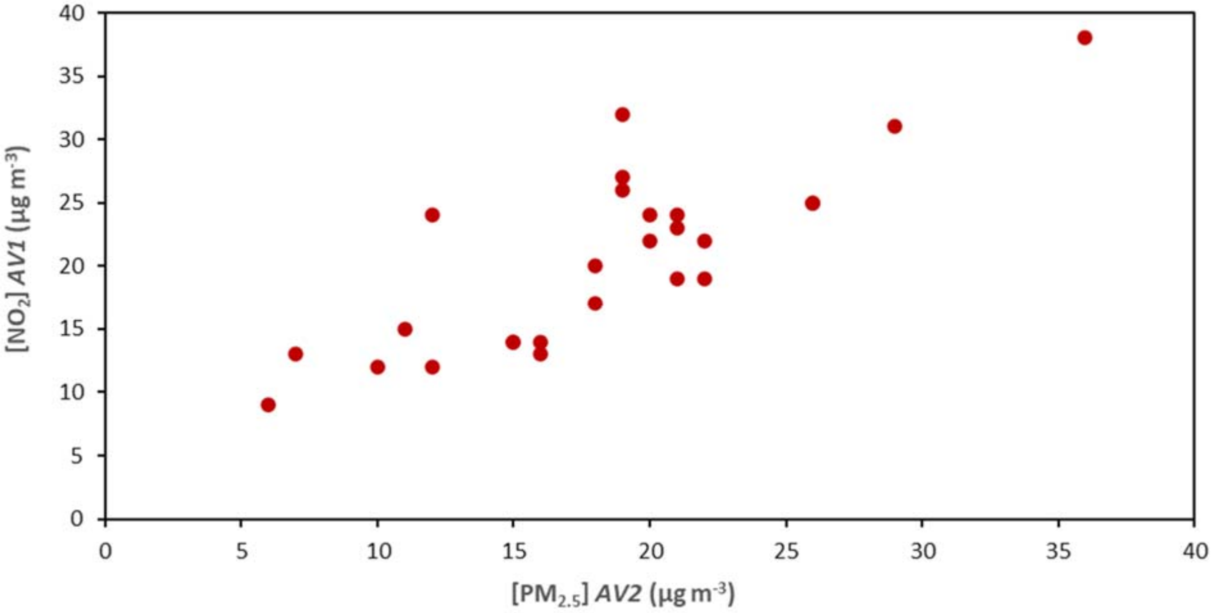
[NO_2] *AV1* (µg m^{-3})
[$PM_{2.5}$] *AV2* (µg m^{-3})

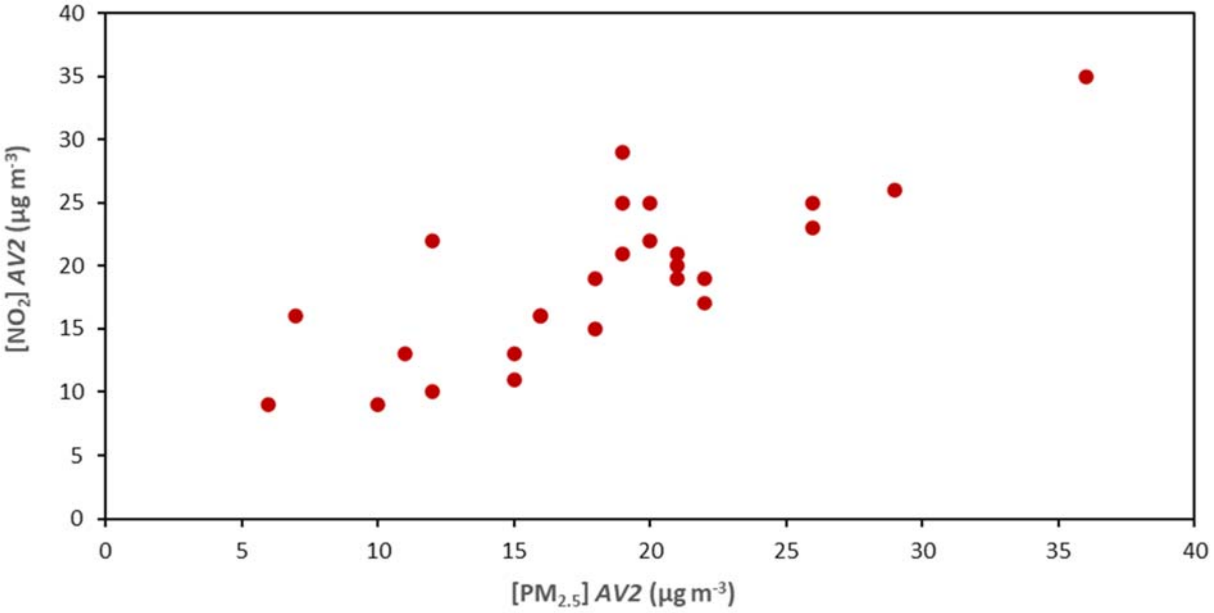
[NO_2] *AV2* (µg m^{-3})
[$PM_{2.5}$] *AV2* (µg m^{-3})

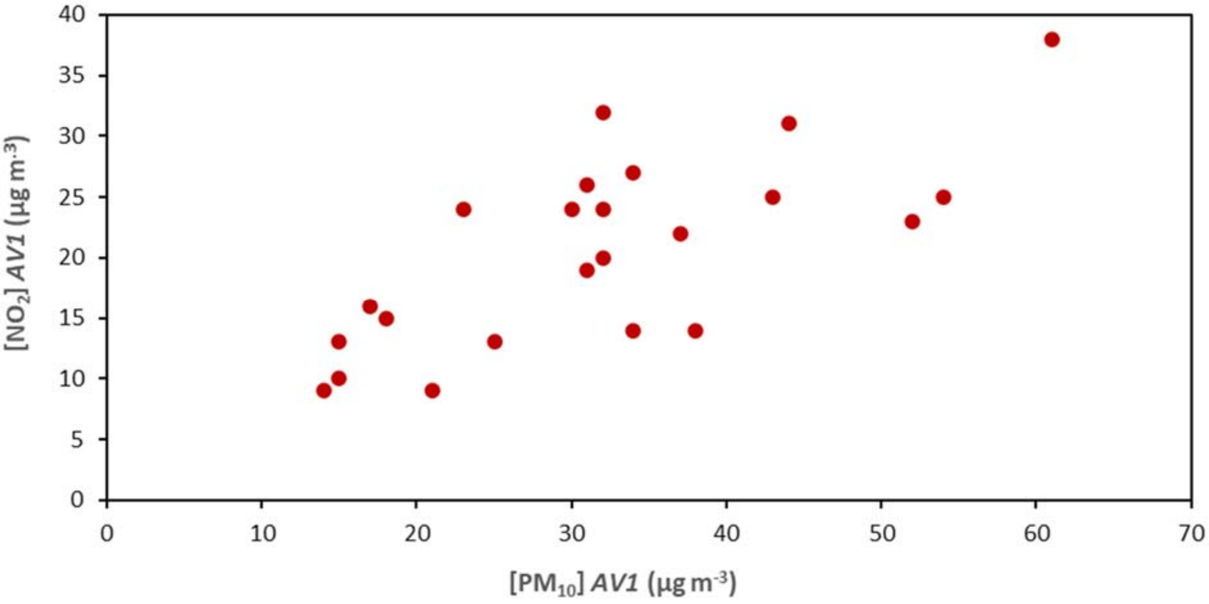

[NO_2] *AV1* (µg m-3)
0
5
10
15
20
25
30
35
40
0
10
20
30
40
50
60
70
[PM_{10}] *AV1* (µg m-3)

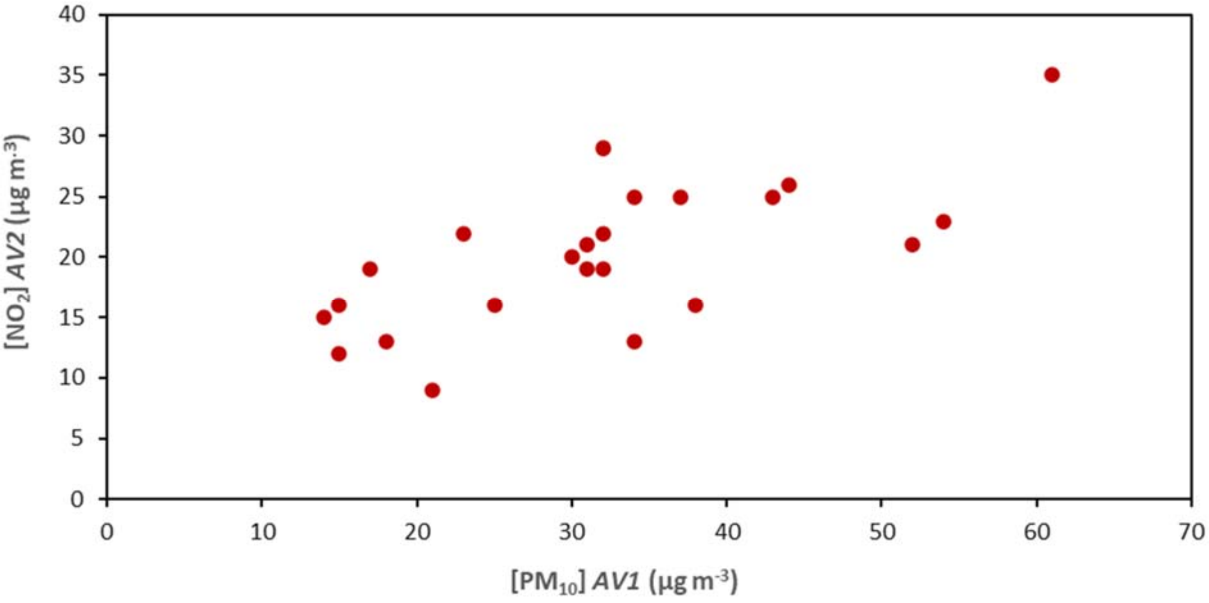

[NO_2] *AV2* (µg m-3)
0
5
10
15
20
25
30
35
40
0
10
20
30
40
50
60
70
[PM_{10}] *AV1* (µg m-3)

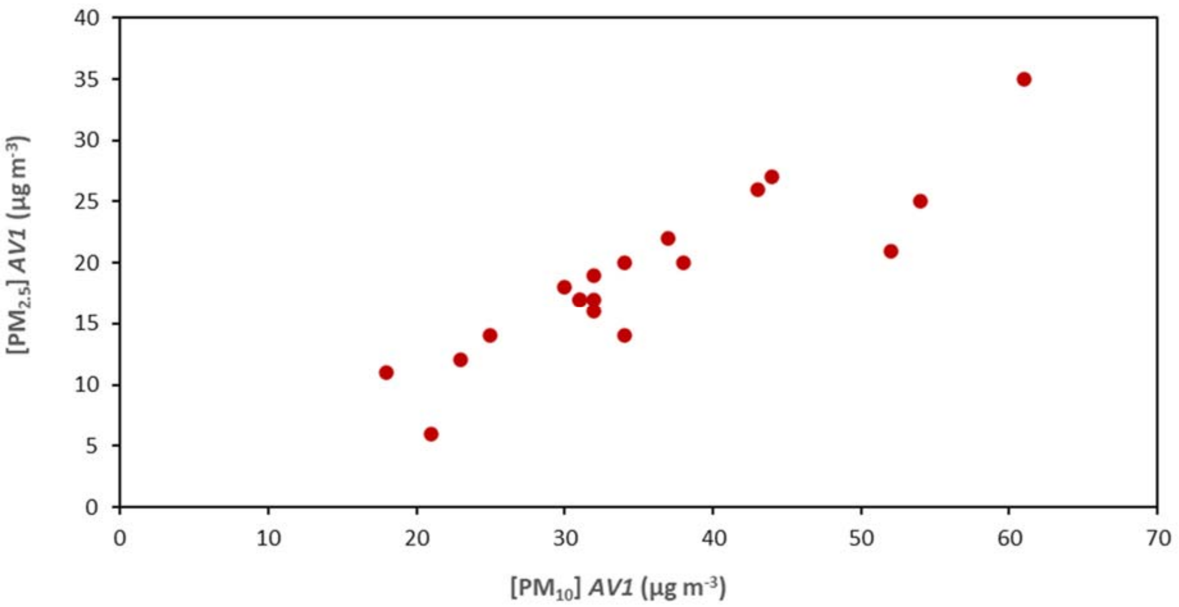

[PM$_{2.5}$] AV1 (µg m^{-3})
[PM$_{10}$] AV1 (µg m^{-3})

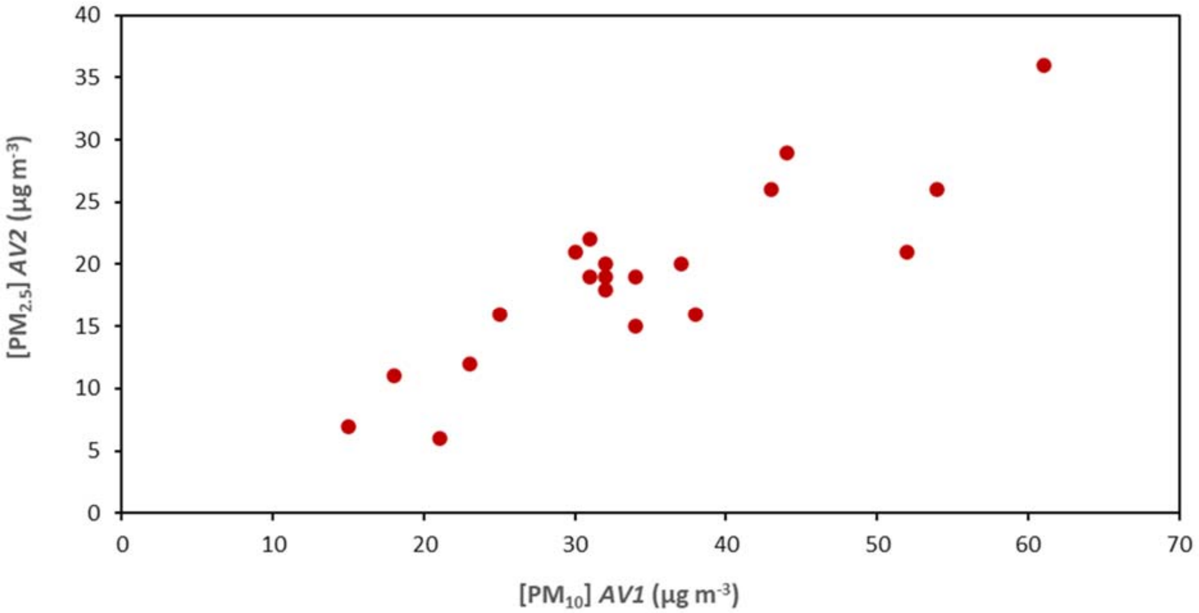

[PM$_{2.5}$] AV2 (µg m^{-3})
[PM$_{10}$] AV1 (µg m^{-3})

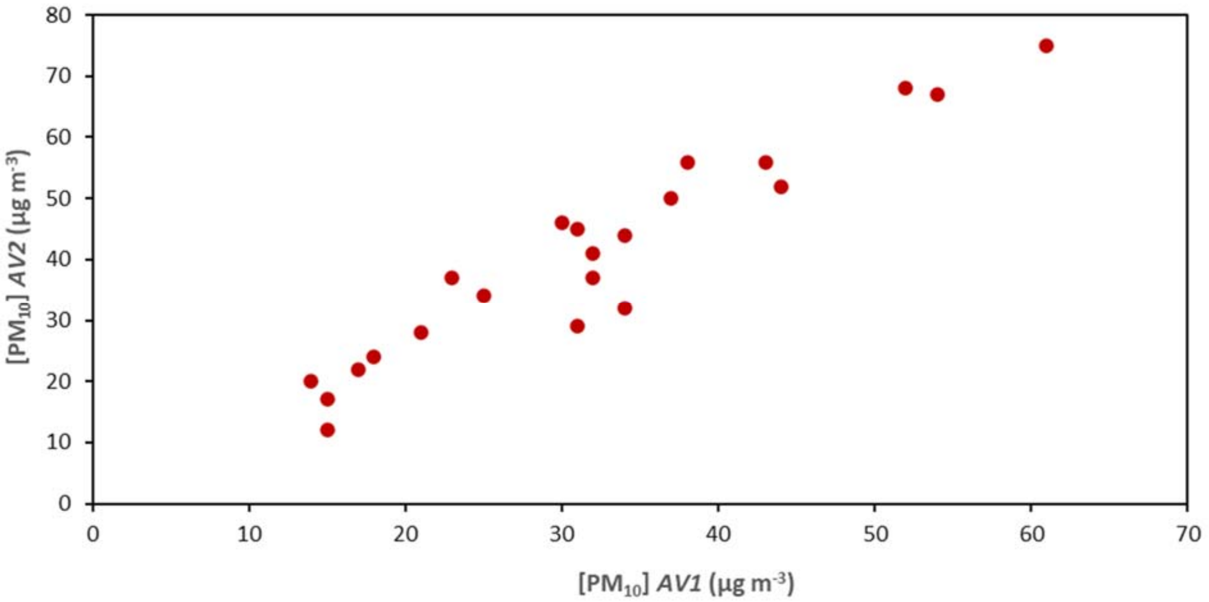
[PM10] AV2 (µg m-3)
[PM10] AV1 (µg m-3)
0
10
20
30
40
50
60
70
80

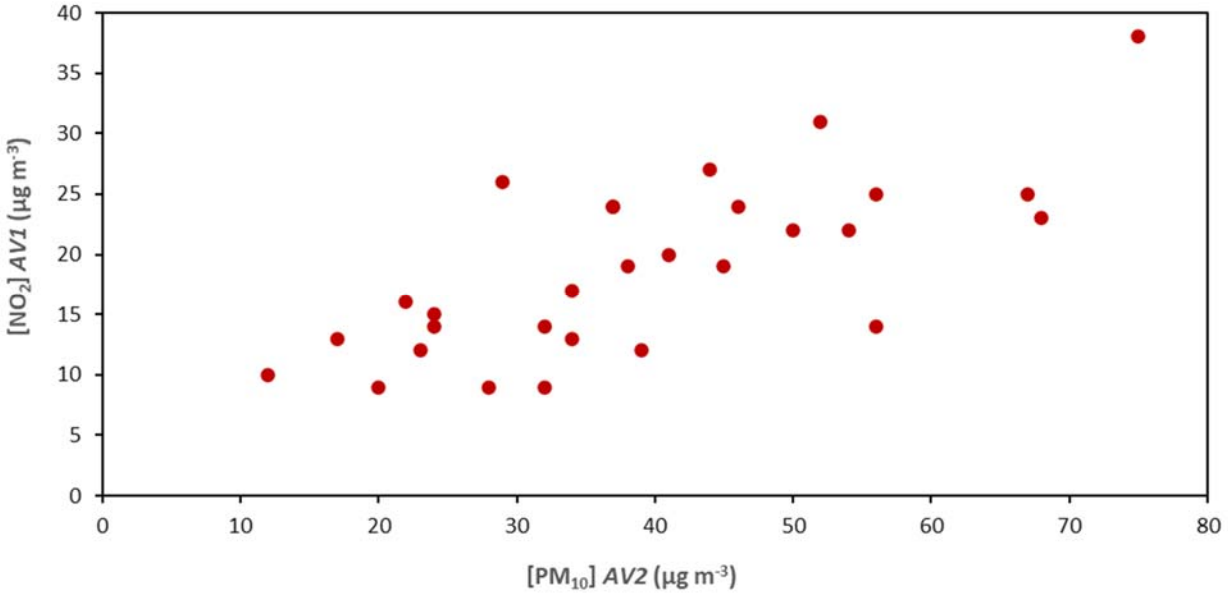
[NO2] AV1 (µg m-3)
[PM10] AV2 (µg m-3)
0
5
10
15
20
25
30
35
40
50
60
70
80

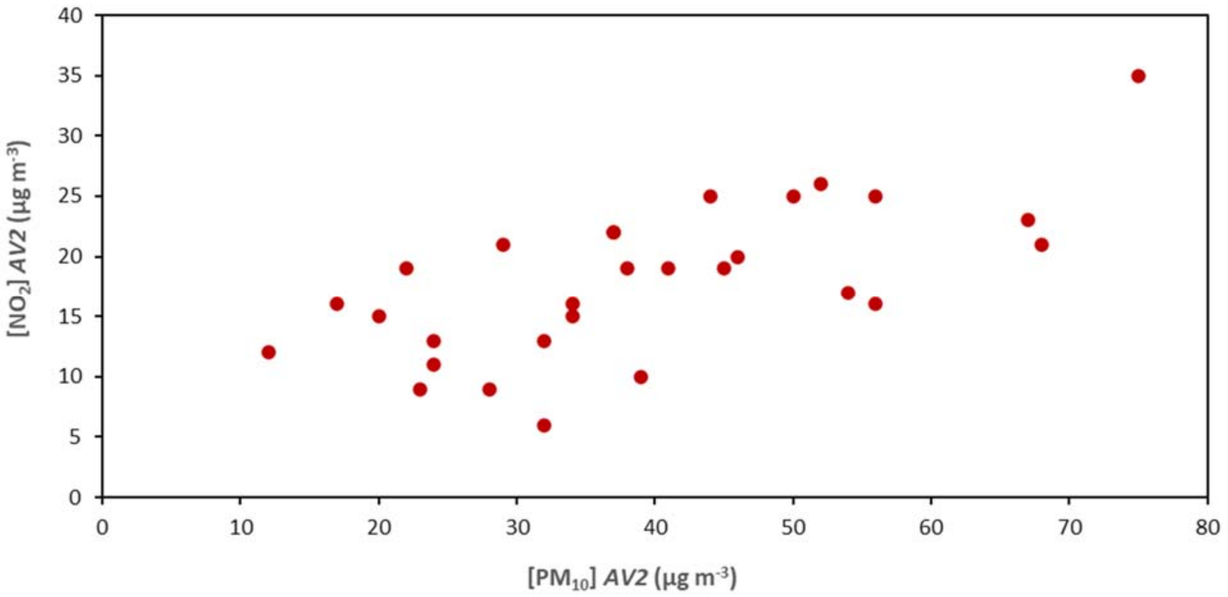

$[NO_2]$ *AV2* ($\mu g\ m^{-3}$)
$[PM_{10}]$ *AV2* ($\mu g\ m^{-3}$)

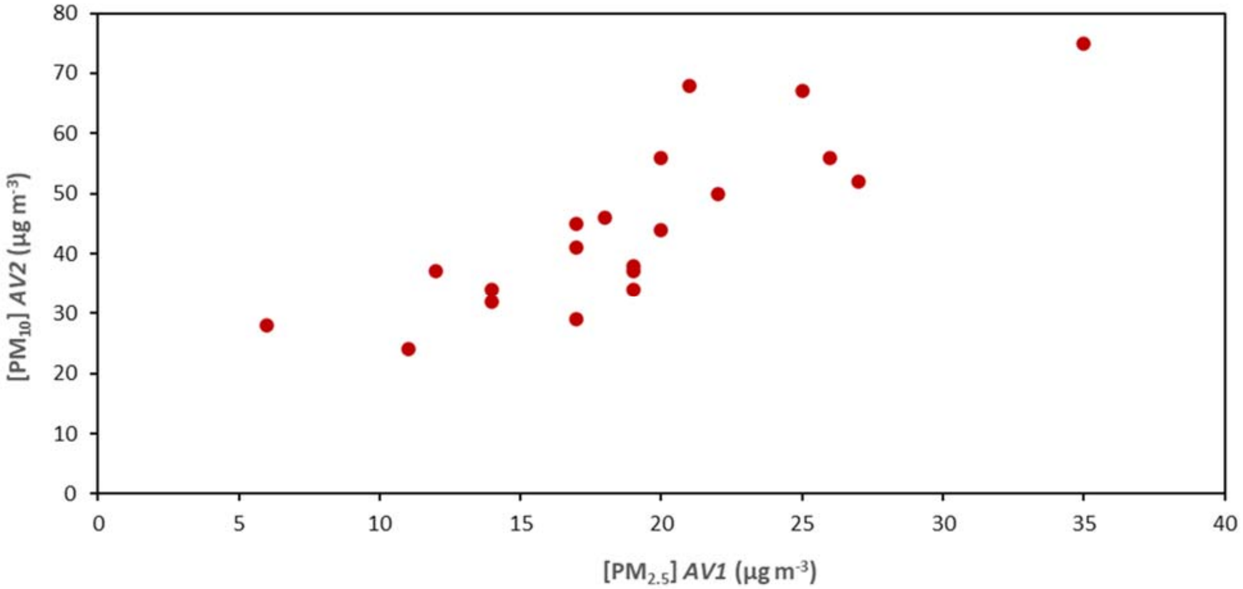

$[PM_{10}]$ *AV2* ($\mu g\ m^{-3}$)
$[PM_{2.5}]$ *AV1* ($\mu g\ m^{-3}$)

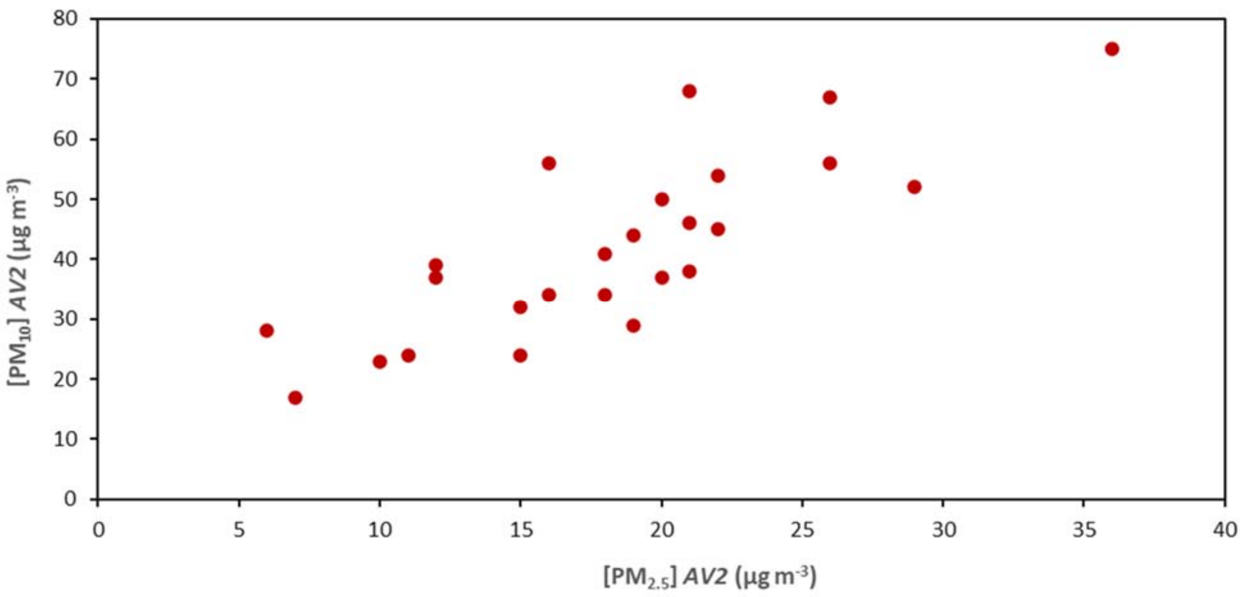

Figure S11. Correlations between the investigated pollutants (period September 1st – September 30st).

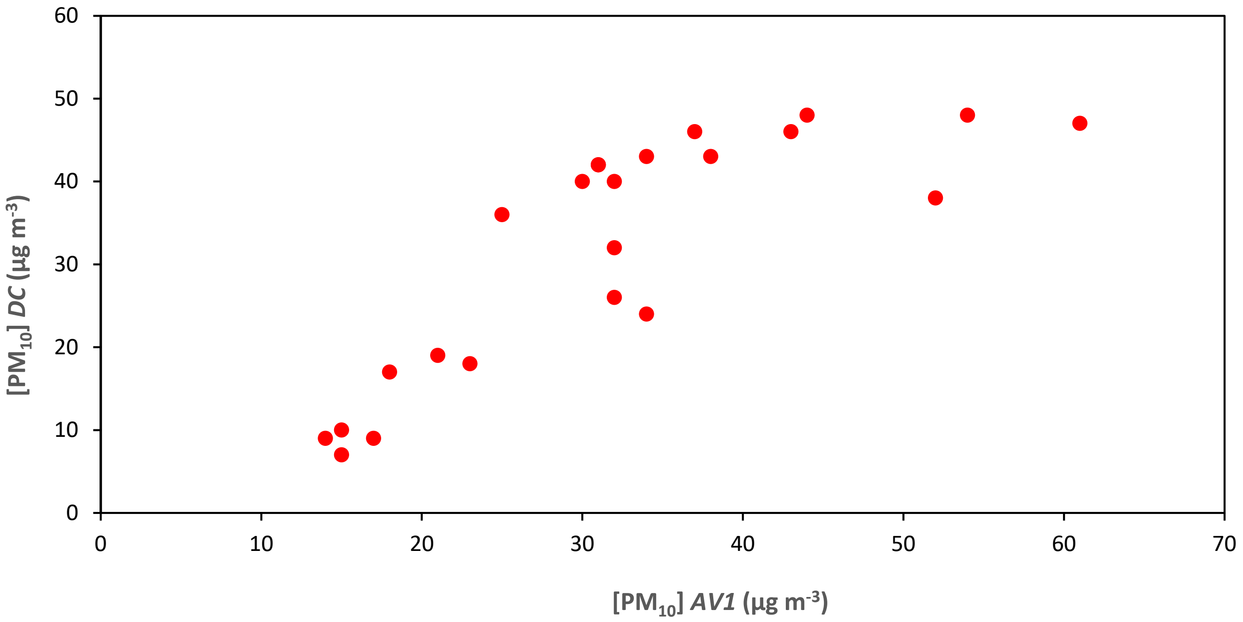

Figure S12. Correlations between [PM10] at AV1 and AURA VDC (period September 1st – September 30st).
